# Evaluation of a Fuzzy-Supervised PID Controller for a Parallel Rehabilitation Mechanism

**DOI:** 10.3390/bioengineering13091029

**Published:** 2026-09-03

**Authors:** Adriana-Daniela Banyai, Daniel-Vasile Banyai, Cornel Brisan

**Affiliations:** 1 Department of Mechatronics and Machine Dynamics, Technical University of Cluj-Napoca, 400114 Cluj-Napoca, Romania; adriana.tomsa@campus.utcluj.ro (A.-D.B.); cornel.brisan@mdm.utcluj.ro (C.B.); 2 Department of Mechanical Engineering, Technical University of Cluj-Napoca, 400114 Cluj-Napoca, Romania

**Keywords:** rehabilitation robotics, parallel mechanism, fuzzy-supervised PID control, actuator-space tracking, Simscape Multibody, CAD-derived dynamics, robustness analysis

## Abstract

Accurate actuator-space tracking is an important engineering requirement for repeatable motion delivery by parallel rehabilitation mechanisms, but controller performance should also be assessed under configuration-dependent dynamics and modeling uncertainty. This study evaluates a bounded fuzzy-supervised proportional–integral–derivative (FSPID) controller for a three-chain 3STC+S parallel rehabilitation mechanism. CAD-derived inverse kinematics generated the three prismatic-joint reference trajectories, while closed-loop behavior was simulated using a Simscape Multibody model including rigid-body mass and inertia properties, gravity, closed-loop constraints, and external force/moment loading. Three fixed-gain PID loops formed the baseline. The FSPID supervisor used zero-order Sugeno inference to adjust proportional and derivative gains within prescribed bounds, while the integral action remained fixed and was implemented with a leaky integrator. Under the nominal 1° trajectory at 0.2 Hz, FSPID reduced mean root-mean-square error (RMSE), maximum absolute error, and integral absolute error (IAE) by 0.25%, 0.37%, and 0.21%, respectively. Under sustained external loading, maximum-error reductions reached 9.33–10.20%, with mean actuator-wise peak-force changes within approximately ±2%. Additional simulations examined trajectory amplitude, synthetic measurement noise, viscous damping, and combined nonidealities. The results support FSPID as a conservative simulation-level refinement of fixed-gain PID; experimental validation remains necessary.

## 1. Introduction

Physical rehabilitation frequently requires intensive, repetitive, and task-specific movement practice to promote motor recovery or functional compensation. Conventional therapy remains indispensable because clinical reasoning, manual assistance, patient motivation, and the adaptation of exercises to individual needs cannot be replaced by an automated system. Nevertheless, rehabilitation robots can complement therapist-led interventions by delivering repeatable movements, maintaining prescribed training conditions, and recording objective information about patient and device performance [1,2]. Evidence from rehabilitation research indicates that the effects of robot-assisted therapy depend on the type of device, the target population, the training protocol, and the manner in which robotic assistance is integrated into the overall therapeutic program [1,3]. Consequently, trajectory-tracking performance should be regarded as a necessary engineering condition for controlled movement delivery rather than as direct evidence of clinical effectiveness.

The design of a rehabilitation robot involves a close relationship between mechanical architecture, actuation, sensing, control, and human–robot interaction. A mechanism may provide a suitable range of motion and load-bearing capacity, yet remain unsuitable for therapeutic use if its motion cannot be controlled accurately and consistently. Conversely, high tracking accuracy obtained through excessive actuator commands may result in an undesirable control solution, particularly in systems intended for physical interaction. Rehabilitation-oriented controllers must therefore balance tracking precision, dynamic stability, command smoothness, actuator limitations, and robustness to changes in operating conditions. Current control approaches range from conventional proportional–integral–derivative (PID) and proportional–derivative controllers to impedance, admittance, assist-as-needed, adaptive, model-based, and intelligent control strategies [4]. Although these approaches address different interaction objectives, their practical implementation must ultimately account for both motion performance and the demand imposed on the actuation system.

PID control remains relevant in rehabilitation robotics because of its simple structure, low computational requirements, and direct physical interpretation. It can be implemented independently at the actuator level and integrated into real-time control architectures without requiring a complete high-fidelity model of the robot. Optimized PID controllers have demonstrated suitable trajectory-tracking performance in rehabilitation mechanisms when the operating conditions remain sufficiently close to those used during controller tuning [5]. However, constant gains impose a fixed compromise among response speed, tracking accuracy, damping, and command effort. A conservative gain set may provide stable and smooth behavior but become less effective when the reference trajectory varies more rapidly or when the system is disturbed. Increasing the gains can improve tracking under demanding conditions, but may also amplify command peaks, oscillations, measurement noise, or sensitivity to modeling uncertainty.

These control requirements are particularly important for parallel rehabilitation mechanisms. Unlike serial manipulators, parallel mechanisms connect the fixed base and moving platform through several kinematic chains. Their closed-loop structure can provide favorable stiffness, load distribution, compactness, and repeatability, but it also introduces coupled kinematics, restricted workspaces, singular configurations, and nonlinear actuator–platform relationships. Parallel mechanisms have therefore been investigated for the rehabilitation of the ankle, lower limb, shoulder, wrist, and other anatomical regions requiring controlled multiaxial movement [6,7,8]. Control evaluation cannot treat the actuators as mechanically independent axes, even when decentralized actuator-level loops are used, because the displacement and loading of each actuator contribute to the common platform motion.

The mechanism considered in the present study was selected during a preceding stage of the research, in which four candidate parallel architectures were compared using kinematic and dynamic modeling, numerical simulation, and multicriteria evaluation [9]. The selected architecture combines three identical peripheral chains with a central spherical coupling and is referred to in the original research notation as 3STC+S; its joint sequence and mobility are defined explicitly in Section 2.1. The preceding study addressed mechanical configuration selection, whereas the present work addresses the subsequent control-design problem. Accordingly, the current contribution does not repeat the comparative mechanical analysis, but uses the selected architecture as the basis for formulating and evaluating an actuator-space controller.

Fuzzy logic provides a practical means of introducing gain adaptation while retaining the transparent structure of PID control. A fuzzy supervisor can modify selected controller gains according to qualitative information derived from the instantaneous tracking state. Previous studies have used fuzzy control and fuzzy adaptation in rehabilitation devices to improve trajectory tracking, regulate human–robot interaction, or adjust assistance according to changes in system behavior [10,11,12,13]. However, improved tracking alone is insufficient to establish that an adaptive controller is preferable for a rehabilitation-oriented mechanism. A supervisory strategy may reduce tracking error by substantially increasing the magnitude or variability of the control command. For this reason, controller assessment should include command-related indicators in addition to conventional error measures.

A further methodological concern is that a supervised controller may appear effective when evaluated only under the nominal conditions used during controller design. Parallel rehabilitation mechanisms are inherently configuration-dependent and may also be affected by external interaction loads, measurement uncertainty, and unmodeled joint resistance. Accordingly, the evaluation considers not only nominal CAD-derived motion but also changes in trajectory amplitude, externally applied forces and moments, synthetic measurement noise, parametric viscous joint damping, and a combined noise–damping condition. In every matched comparison, the fixed-gain PID and fuzzy-supervised PID controllers operate on the same CAD-derived multibody plant, use identical reference trajectories and initial conditions, and are evaluated on a common numerical grid. This protocol is intended to distinguish the effect of fuzzy supervision from changes in plant or test conditions while providing a broader simulation-based assessment of robustness.

The objective of this study is to formulate and evaluate a conservative fuzzy-supervised PID controller for the selected three-chain 3STC+S parallel rehabilitation mechanism using a CAD-derived rigid-body multibody simulation plant. The control problem is formulated in actuator space, while the prescribed platform orientation motion is converted into three prismatic-joint reference trajectories through inverse kinematics of the CAD-derived mechanism. Closed-loop forward dynamics are simulated in Simscape Multibody using the rigid-body geometry, mass and inertia properties, gravity, closed-loop mechanical constraints, and externally applied force/torque inputs. Three decentralized fixed-gain PID loops provide the baseline controller. The proposed zero-order Sugeno supervisor adjusts only the proportional and derivative gains from normalized tracking error and error rate, whereas the integral contribution uses a fixed gain with a leaky integrator to limit long-term accumulation under sustained loading. All controller and fuzzy-supervisor parameters are held fixed throughout the comparative simulation protocol.

The main contributions of this study are: (i) replacement of the previously used reduced actuator-space plant by a CAD-derived Simscape Multibody model of the complete 3STC+S mechanism, thereby preserving the configuration-dependent rigid-body dynamics and closed-loop mechanical constraints; (ii) generation of actuator-space reference trajectories through inverse kinematics of the same CAD-derived mechanism; (iii) formulation of a bounded and interpretable zero-order Sugeno supervisor that adjusts the proportional and derivative gains while retaining fixed-gain leaky integral action; (iv) a matched PID–FSPID comparison under nominal motion, external force and moment disturbances, synthetic measurement noise, parametric viscous damping, combined nonidealities, and trajectory-amplitude variation; and (v) explicit reporting of tracking accuracy, actuator-force demand, fuzzy-operating ranges, and numerical consistency checks. The study remains simulation-based; no hardware-in-the-loop, prototype, or clinical validation is claimed.

## 2. Materials and Methods

### 2.1. Mechanism Architecture and Actuator-Reference Generation

The mechanism investigated in this study is the parallel rehabilitation architecture selected during the preceding mechanical-design stage [9]. It comprises three identical peripheral kinematic chains and a central spherical coupling between the fixed base and the moving platform. In the notation adopted herein, the mechanism is denoted by 3STC+S. The prefix ‘3′ indicates the three identical chains, whereas STC denotes the sequence spherical joint–actuated translational joint–cardan joint within each chain. The additional ‘+S’ represents the central spherical coupling. The three prismatic joints are the active coordinates of the mechanism.

**Figure 1:**
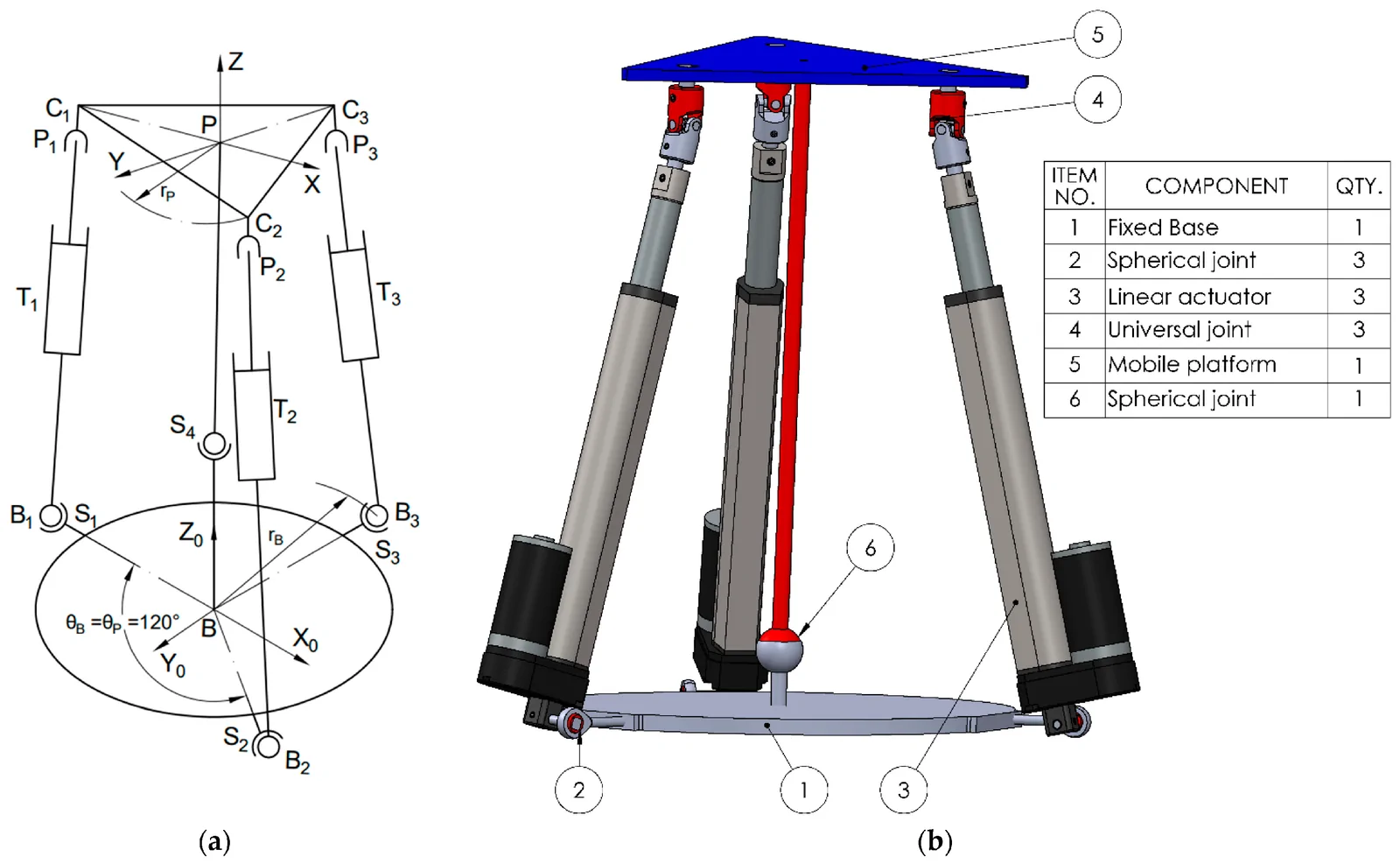
Three-chain parallel rehabilitation mechanism with a central spherical coupling: (**a**) kinematic interpretation; (**b**) CAD configuration and component identification. Adapted from Todor et al. [9].

The central spherical coupling constrains translation of the platform reference point relative to the base while permitting rotation about the coupling center. Coordinated changes in the three peripheral active-chain lengths therefore generate the prescribed roll, pitch, and yaw motion of the platform, subject to chain-length, workspace, and singularity constraints. The controller is implemented in actuator space, whereas the rehabilitation-oriented motion is specified initially as a platform-orientation trajectory. The architecture and its three-rotational-degree-of-freedom interpretation are shown in Figure 1.

The actuator-space references used in the study were generated directly from the CAD-derived multibody representation rather than from a reduced analytical chain-length approximation. The assembled CAD configuration was imported into Simscape Multibody, and the three prismatic-joint coordinates were defined relative to the verified neutral configuration, for which *q_1_ = q_2_ = q_3_ = 0*. The neutral platform pose corresponds to the assembled CAD configuration and was not redefined as a zero-Euler-angle pose. A Simscape Multibody Kinematics Solver was first used to obtain a kinematically consistent neutral state of the closed-loop mechanism. This state was then used as the initial solution for continuation along the same inverse-kinematic branch during the prescribed motion.

Platform motion was prescribed for the moving-platform Rotation.y coordinate. For the nominal case, the angular increment relative to the neutral CAD orientation was defined as:

(1) $$\Delta \theta_{y}\left(t\right)=Asin\left(2\pi ft\right)$$

where *A* = 1.0° and *f* = 0.2 Hz. At each time sample, the complete inverse-kinematic problem of the closed-loop multibody mechanism was solved for the three active prismatic coordinates. The solution obtained at the preceding time step was used to initialize the subsequent inverse-kinematic solution, thereby maintaining continuity on the same kinematic branch. The resulting actuator-reference vector was:

(2) $$q_{ref}\left(t\right)=\left[\begin{array}{c} q_{1,ref}\left(t\right)\\ q_{2,ref}\left(t\right)\\ q_{3,ref}\left(t\right) \end{array}\right]$$

All actuator-reference trajectories were generated using a sampling interval of *Ts* = 0.002 s over a duration of 5 s, resulting in 2501 samples per trajectory. Reference velocities and accelerations were calculated from the solved periodic actuator-coordinate histories using central finite differences. For sample k, we have the following:

(3) $$\dot{q_{ref,k}}=\frac{q_{ref,k+1}-q_{ref,k-1}}{2T_{s}}$$

and

(4) $$\ddot{q_{ref,k}}=\frac{q_{ref,k+1}-2q_{ref,k}+q_{ref,k-1}}{{T_{s}}^{2}}$$

with periodic indexing applied at the trajectory boundaries.

For the trajectory-amplitude sensitivity analysis, the same inverse-kinematic procedure, excitation frequency, sampling interval, duration, and branch-continuity strategy were retained while the Rotation.y amplitude was set to 0.5°, 1.0°, and 1.5°. The reference-generation settings are summarized in Table 1.

Numerical consistency of the reference-generation procedure was checked for every inverse-kinematic sample. All 2501 configurations were solved successfully for each investigated amplitude. The maximum actuator-vector increment between consecutive samples increased from 0.004738 mm at 0.5° to 0.014214 mm at 1.5°, without discontinuities indicative of branch switching. Periodic closure errors remained below 6 × 10^−12^ mm for all three trajectories. PID and FSPID therefore received exactly the same CAD-derived actuator-reference trajectory in each matched comparison.

### 2.2. CAD-Derived Simscape Multibody Dynamic Plant

**Figure 2:**
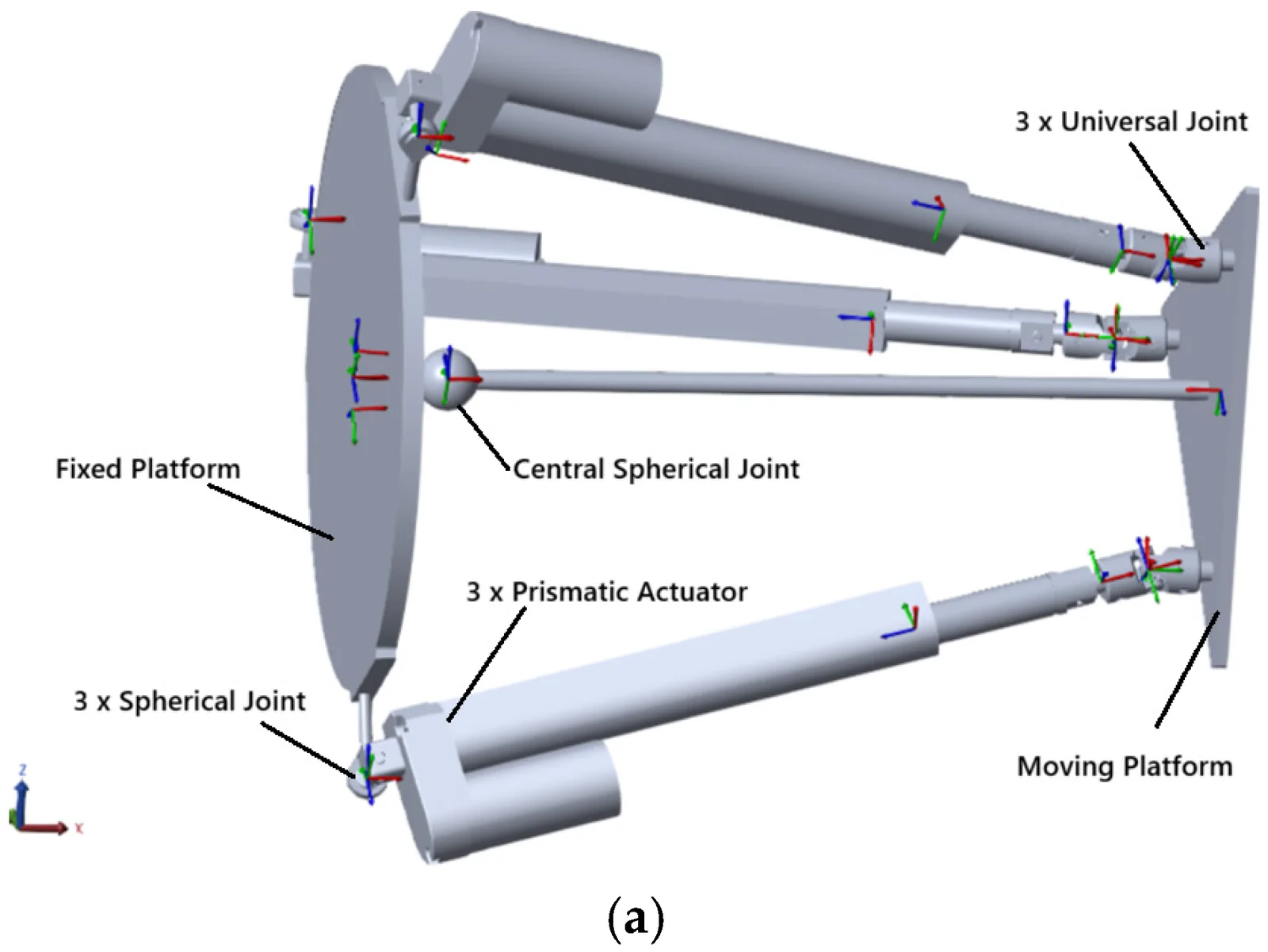

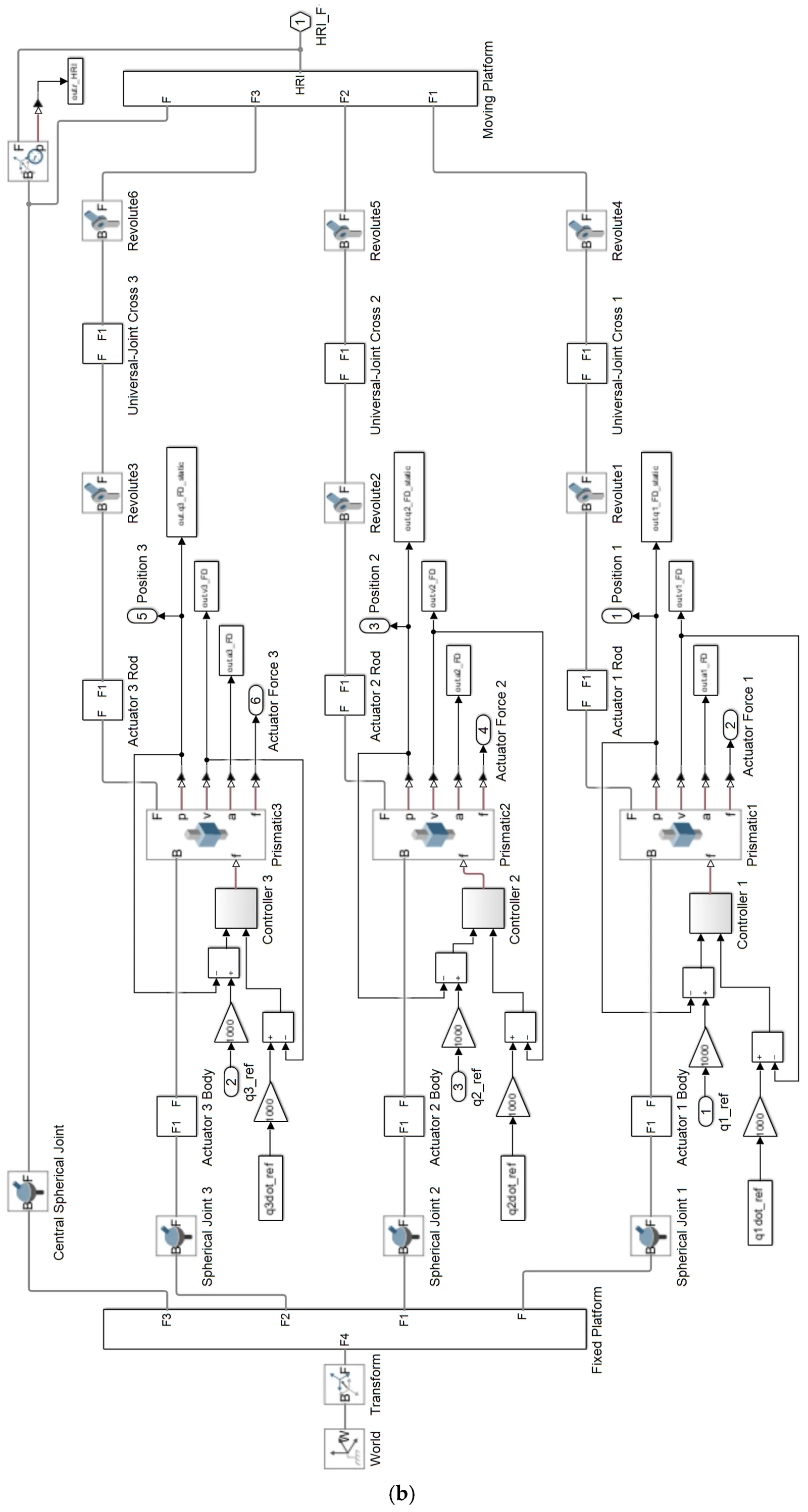

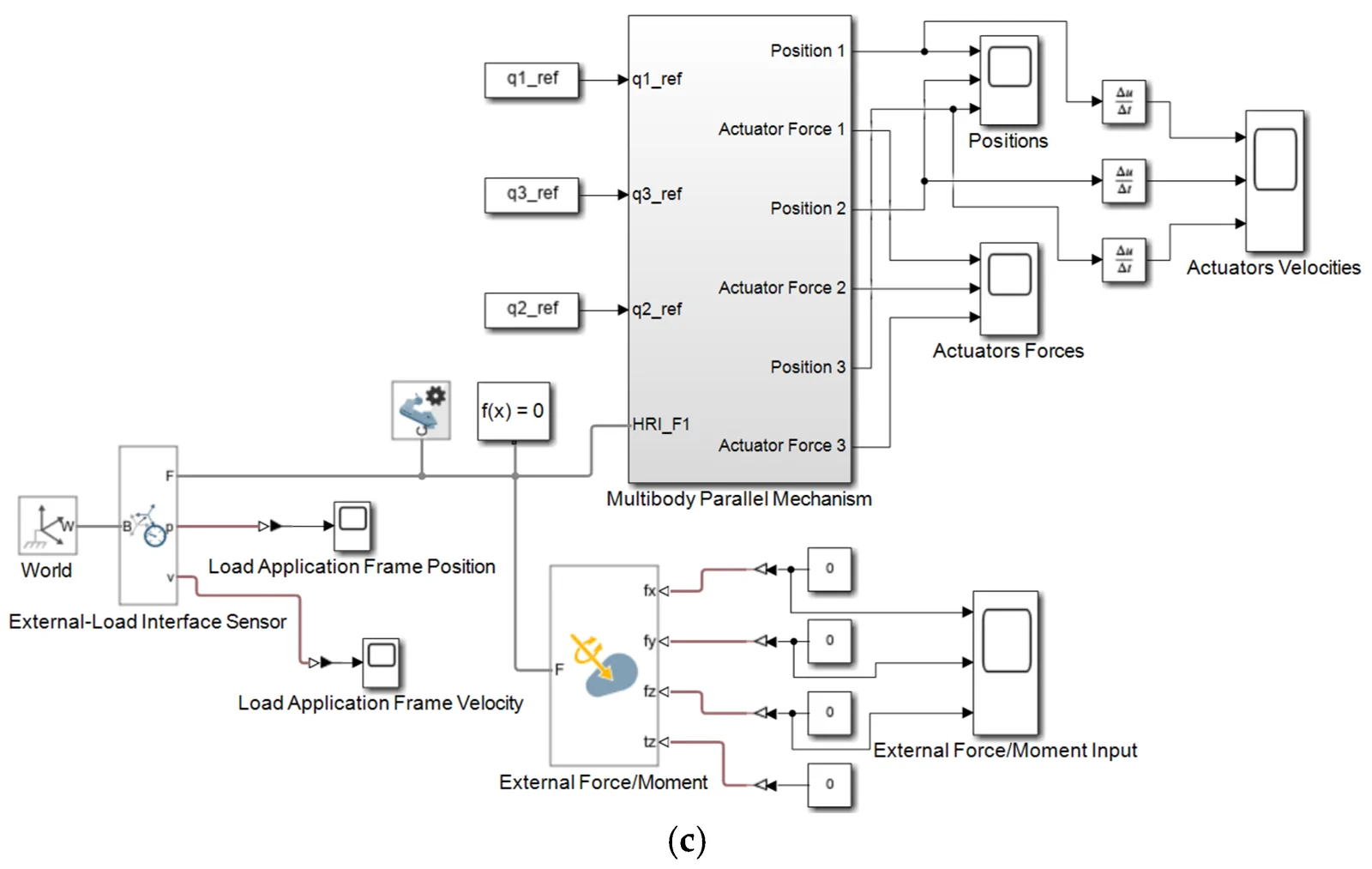
CAD-derived simulation plant and workflow: (**a**) 3STC+S rigid-body assembly visualized in Simscape Multibody; (**b**) closed-loop multibody subsystem linking CAD-derived actuator references and PID/FSPID control; (**c**) top-level Simulink interface for externally applied forces and moments.

Closed-loop forward dynamics were evaluated using a CAD-derived mechanism-level rigid-body model of the assembled 3STC+S mechanism in MATLAB/Simulink R2025a with Simscape Multibody (MathWorks, Natick, MA, USA). The complete CAD-derived simulation model and workflow are presented in Figure 2. The multibody plant was generated from the imported CAD assembly and retained the rigid transforms, joint topology, centers of mass, moments of inertia, and products of inertia contained in the CAD-derived Simscape Multibody data. Consequently, mechanical coupling among the three actuator chains is generated by the closed-loop mechanism itself rather than by an imposed constant inter-actuator coupling coefficient.

The model contains three repeated actuator bodies, three actuator rods, three universal joints, the fixed platform, and the moving platform. The three active coordinates are the prismatic-joint displacements, with the verified neutral CAD configuration defined by *q1 = q2 = q3 = 0*. The remaining revolute and spherical joints preserve the passive-joint topology of the mechanism. Simscape Multibody resolves the closed kinematic loops through its algebraic constraint equations.

For compact notation, the constrained rigid-body dynamics solved numerically by Simscape Multibody can be represented as:

(5) $$M\left(q\right)\ddot{q}+h\left(q,\dot{q}\right)+{\Phi_{q}}^{T}\lambda_{c}=BF_{cmd}+{J_{w}}^{T}W_{ext}$$

subject to the closed-loop constraint equations

(6) $$\Phi \left(q\right)=0$$

where *q* denotes the generalized multibody coordinates, *M*(*q*) is the configuration-dependent mass matrix, *h* collects velocity-dependent and gravitational terms, *Φq* is the constraint Jacobian, and *λc* contains the corresponding constraint reactions. Here, *B* denotes the generalized actuation mapping associated with the three prismatic force inputs, while *JwT*(*q*)—the transpose of the load-frame Jacobian *Jw*(*q*)—maps the external wrench applied at the moving-platform load frame into generalized forces. The three commanded prismatic-joint forces are:

(7) $$F_{cmd}=\left[\begin{array}{c} F_{1}\\ F_{2}\\ F_{3} \end{array}\right]$$

and the external wrench applied at the CAD-defined moving-platform frame is

(8) $$W_{ext}=\left[\begin{array}{c} F_{x}\\ F_{y}\\ F_{z}\\ M_{x}\\ M_{y}\\ M_{z} \end{array}\right]$$

The matrices *M*(*q*), *Φq*(*q*), and *Jw*(*q*) therefore vary with the instantaneous mechanism configuration and are not replaced by a constant Jacobian or a constant pairwise coupling matrix. Constraint reactions and load redistribution among the three chains are obtained directly from the multibody solution at each integration step. The controller itself remains actuator-space based and does not require an explicitly linearized mechanism Jacobian.

The CAD-derived inertial mapping was audited before the final simulations. Rigid-body instances were checked against the imported CAD source, and the final model retained the corrected mass, center-of-mass, moment-of-inertia, and product-of-inertia assignments. Table 2 summarizes the principal rigid-body and multibody settings used in the simulations; detailed inertial data are provided in the Supplementary Materials.

Gravity was enabled in every dynamic simulation. The nominal prismatic-joint viscous-damping coefficient was zero because experimentally identified actuator-friction parameters were not available. The native joint damping coefficient was varied only in the dedicated sensitivity study described in Section 2.5; those values are therefore treated as parametric modeling uncertainty rather than measured friction coefficients. Native hard-stop contact parameters were not used as a surrogate friction model.

External loading was introduced through a six-component force/torque block connected to a CAD-defined frame on the moving platform. This interface permits independent force and moment components while preserving the same closed-loop plant for both controllers. The external-load cases are numerical disturbance scenarios intended to examine disturbance rejection and are not presented as experimentally measured patient interaction loads.

As an internal numerical consistency check, inverse- and forward-dynamics calculations were cross-checked at the neutral configuration under gravity and an applied Mz = 1 N·m moment. When the actuator forces obtained from the inverse-dynamics model were applied to the forward-dynamics model under the same loading, the short-duration residual motion remained at the numerical round-off level. This test verifies internal consistency between the inverse- and forward-dynamics formulations of the CAD-derived model but does not constitute experimental validation. Detailed force values and residual-motion data are provided in the Supplementary Materials.

The resulting plant is therefore a mechanism-level rigid-body model rather than an experimentally identified electromechanical prototype model. Motor electrical dynamics, transmission backlash, structural flexibility, and measured actuator friction were not available and are not introduced as fitted parameters.

### 2.3. Fixed-Gain and Fuzzy-Supervised PID Architectures

Three decentralized actuator-space feedback loops were implemented, one for each active prismatic joint. The feedback laws are evaluated independently at the controller level, whereas mechanical coordination and load redistribution arise from the common closed-loop Simscape Multibody plant described in Section 2.2. Tracking error and error rate are expressed in mm and mm/s, respectively, and the controller output is the commanded prismatic-joint force in N.

For actuator *i*, the position-tracking error is:

(9) $$e_{i}\left(t\right)=q_{ref,i}\left(t\right)-q_{i}\left(t\right)$$

and the error-rate signal is

(10) $$\dot{e_{i}}\left(t\right)=\dot{q_{ref,i}}\left(t\right)-\dot{q_{i}}\left(t\right)$$

Both PID and FSPID use leaky integral action rather than a pure integrator. The integral state is governed by:

(11) $$\dot{z_{i}}\left(t\right)=e_{i}\left(t\right)-\lambda z_{i}\left(t\right)$$

which is equivalent to a first-order leaky integrator with transfer function 1/(s + *λ*). The retained value is *λ* = 0.02 s^−1^. This modification was introduced after sustained-load numerical checks showed that pure integral action could accumulate internal preload over long simulations. Reducing Ki slowed this accumulation but did not remove it; therefore, *Ki* was kept fixed, and a small leakage term was introduced. The selected *λ* value is an engineering trade-off obtained from a sensitivity study and is not claimed to be globally optimal.

The fixed-gain PID feedback contribution is:

(12) $$F_{PID,i}=K_{p0}e_{i}+K_{i}z_{i}+K_{d0}\dot{e_{i}}$$

with *Kp0* = 20 N/mm, *Ki* = 2 N/(mm·s), and *Kd0* = 0.5 N·s/mm. Equation (12) defines the feedback contribution. The total actuator command also includes the same constant neutral-configuration gravity-compensation vector for both controllers, *Fg* = [15.221, −13.171, −18.127]^T^ N, obtained from inverse dynamics of the CAD-derived mechanism at *q* = 0. The same *Ki* and leaky-integrator parameter are used by PID and FSPID; the fuzzy supervisor modifies only *Kp* and *Kd*. A common ±100 N numerical limiter is retained as a diagnostic saturation bound for both controllers and is not presented as a verified physical actuator or clinical safety limit.

**Figure 3:**
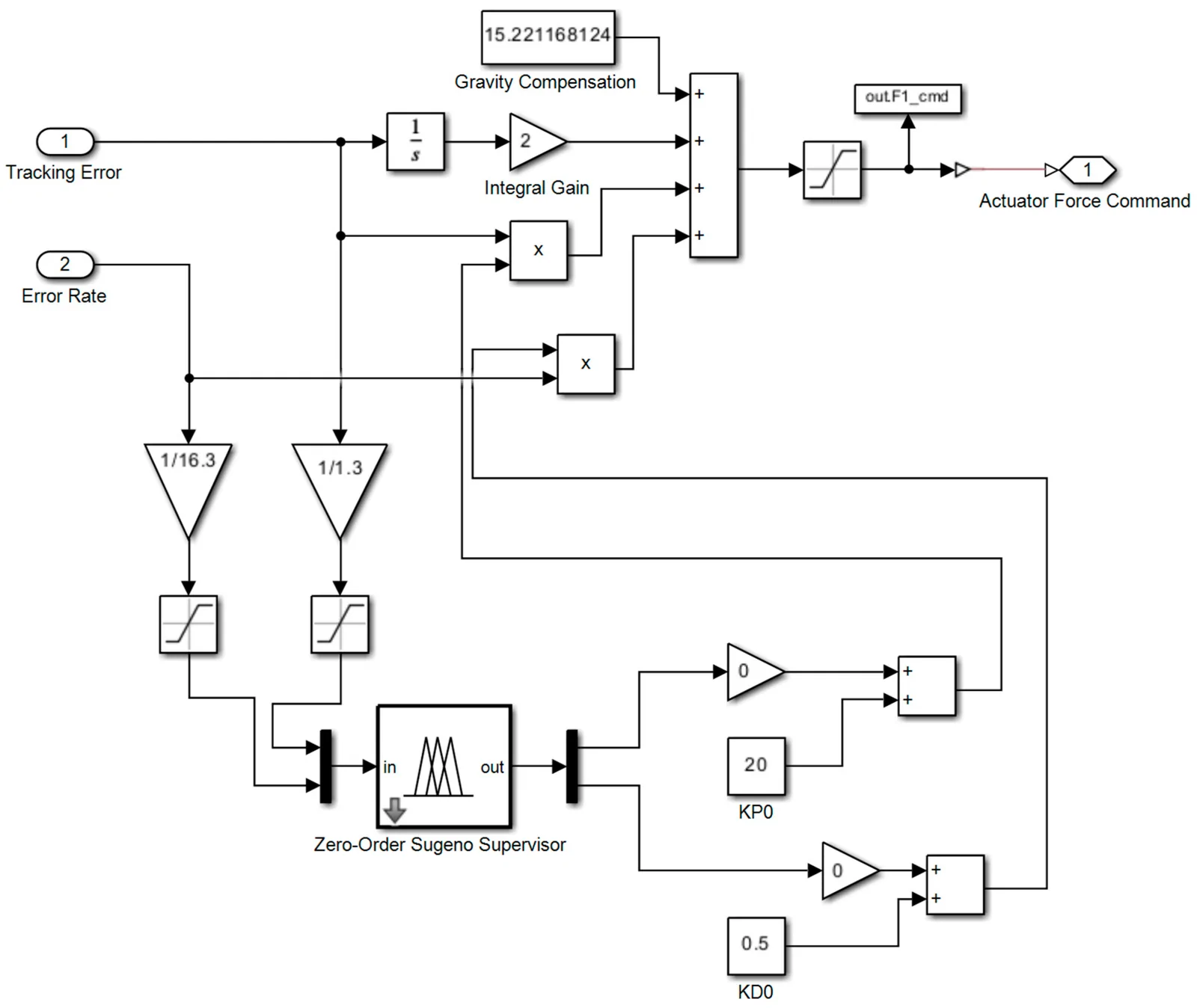
Representative actuator-space control architecture used in the PID–FSPID comparison.

The representative actuator-space control architecture is shown in Figure 3. The same structure is replicated for the three active prismatic joints; the PID baseline corresponds to zero fuzzy gain increments, whereas FSPID uses the supervisor to generate bounded Δ*Kp* and Δ*Kd*.

Keeping the integral channel fixed was deliberate. In the closed-loop parallel mechanism, sustained integral action can alter the internal distribution of actuator forces even when the platform pose changes only slightly. Adapting *Ki* would introduce an additional slow supervisory channel affecting this internal force distribution. Fuzzy supervision is therefore limited to the proportional and derivative terms.

### 2.4. Zero-Order Sugeno Fuzzy Supervisor

The fuzzy supervisor is implemented independently in each actuator loop. Its inputs are the normalized tracking error and normalized error rate, both clipped to the following interval [−1, 1]:

(13) $$e_{N,i}={sat}_{\left[-1,1\right]}\left(\frac{e_{i}}{E_{s}}\right)$$

(14) $$\dot{e_{N,i}}={sat}_{\left[-1,1\right]}\left(\frac{\dot{e_{i}}}{E_{ds}}\right)$$

**Figure 4:**
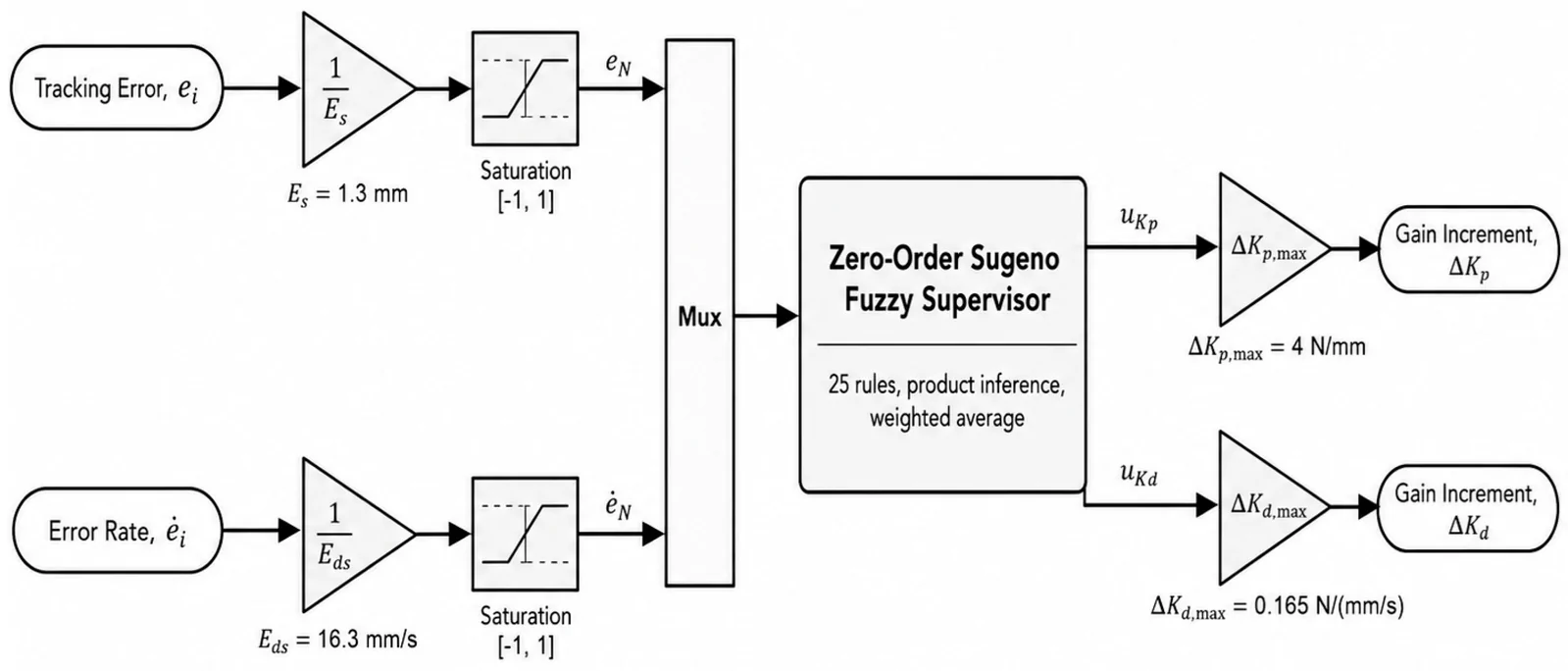
Internal structure of the zero-order Sugeno fuzzy supervisor.

The final scaling constants are *Es* = 1.3 mm and *Eds* = 16.3 mm/s. They were selected from a preliminary audit of the baseline-PID error envelopes so that the audited normalized inputs remained within the prescribed interval and were then frozen before the final PID–FSPID comparisons. They are engineering design values rather than experimentally identified or optimization-derived parameters. The internal structure of the zero-order Sugeno fuzzy supervisor is shown in Figure 4.

**Figure 5:**
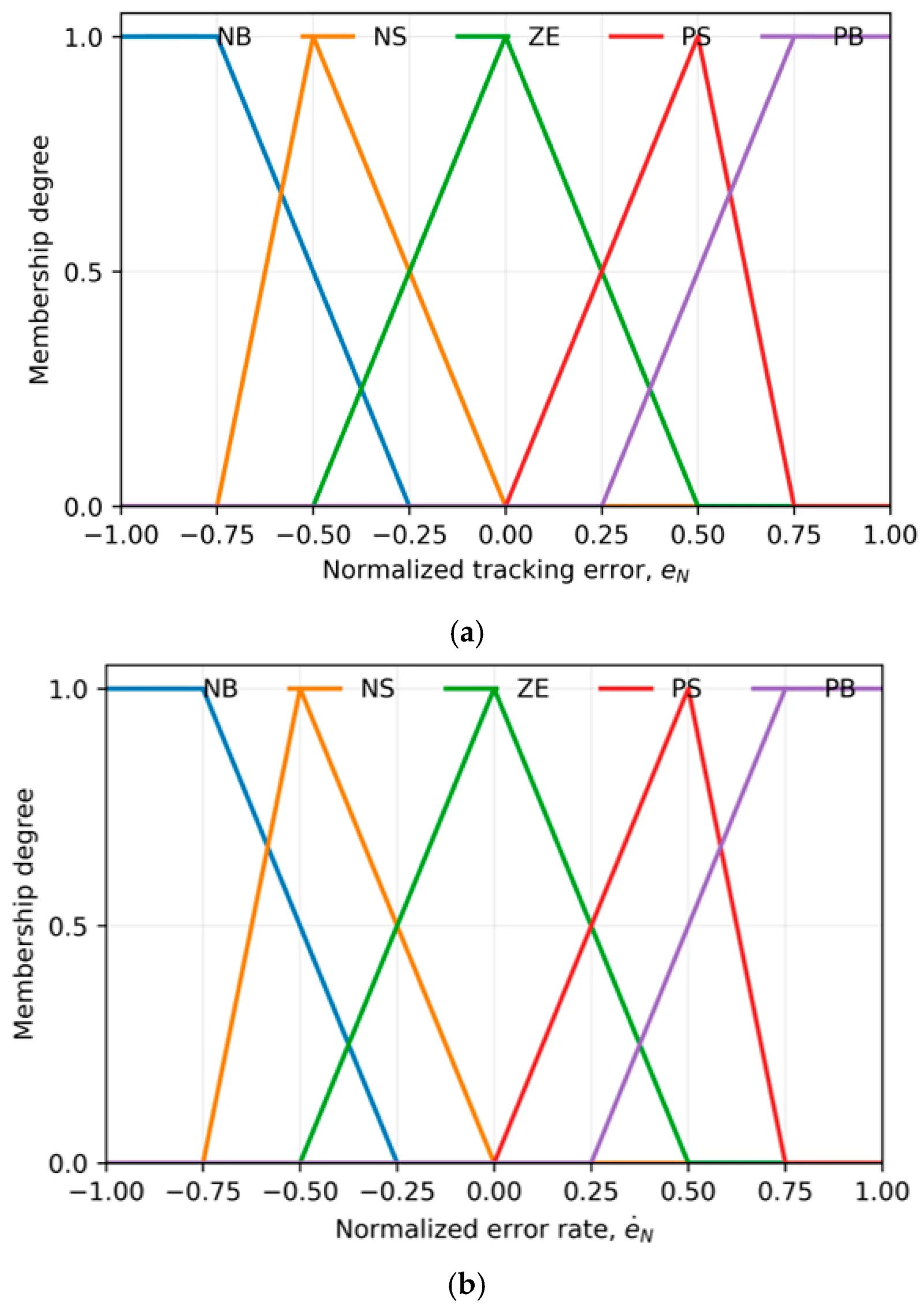
Membership functions used for (**a**) normalized tracking error and (**b**) normalized error rate.

Each normalized input uses five linguistic sets: negative big (NB), negative small (NS), zero (ZE), positive small (PS), and positive big (PB). The membership functions are identical for the two inputs. NB and PB are trapezoidal shoulder functions with parameters [−1, −1, −0.75, −0.25] and [0.25, 0.75, 1, 1], respectively. NS, ZE, and PS are triangular functions with parameters [−0.75, −0.50, 0], [−0.50, 0, 0.50], and [0, 0.50, 0.75], respectively. The corresponding membership functions for the normalized tracking error and normalized error rate are shown in Figure 5.

A zero-order Sugeno inference system with product conjunction is used. For either normalized output *r* ∈ {*Kp*, *Kd*}, the weighted-average output is:

(15) $$u_{r,i}=\frac{\sum_{j=1}^{25}w_{j,i}c_{r,j}}{\sum_{j=1}^{25}w_{j,i}},r\in \{Kp,Kd\}$$

The constant consequents belong to {0, 0.25, 0.50, 0.75, 1.00}. With ordered NB, NS, ZE, PS, and PB along both axes, the normalized proportional-gain and derivative-gain consequent matrices are:

(16) $$U_{Kp}=\left[\begin{array}{ccccc} 1.00 & 0.75 & 0.75 & 0.75 & 1.00\\ 0.50 & 0.50 & 0.25 & 0.50 & 0.50\\ 0.25 & 0 & 0 & 0 & 0.25\\ 0.50 & 0.50 & 0.25 & 0.50 & 0.50\\ 1.00 & 0.75 & 0.75 & 0.75 & 1.00 \end{array}\right]$$

(17) $$U_{Kd}=\left[\begin{array}{ccccc} 1.00 & 0.50 & 0.25 & 0.50 & 1.00\\ 0.75 & 0.50 & 0 & 0.50 & 0.75\\ 0.75 & 0.25 & 0 & 0.25 & 0.75\\ 0.75 & 0.50 & 0 & 0.50 & 0.75\\ 1.00 & 0.50 & 0.25 & 0.50 & 1.00 \end{array}\right]$$

**Figure 6:**
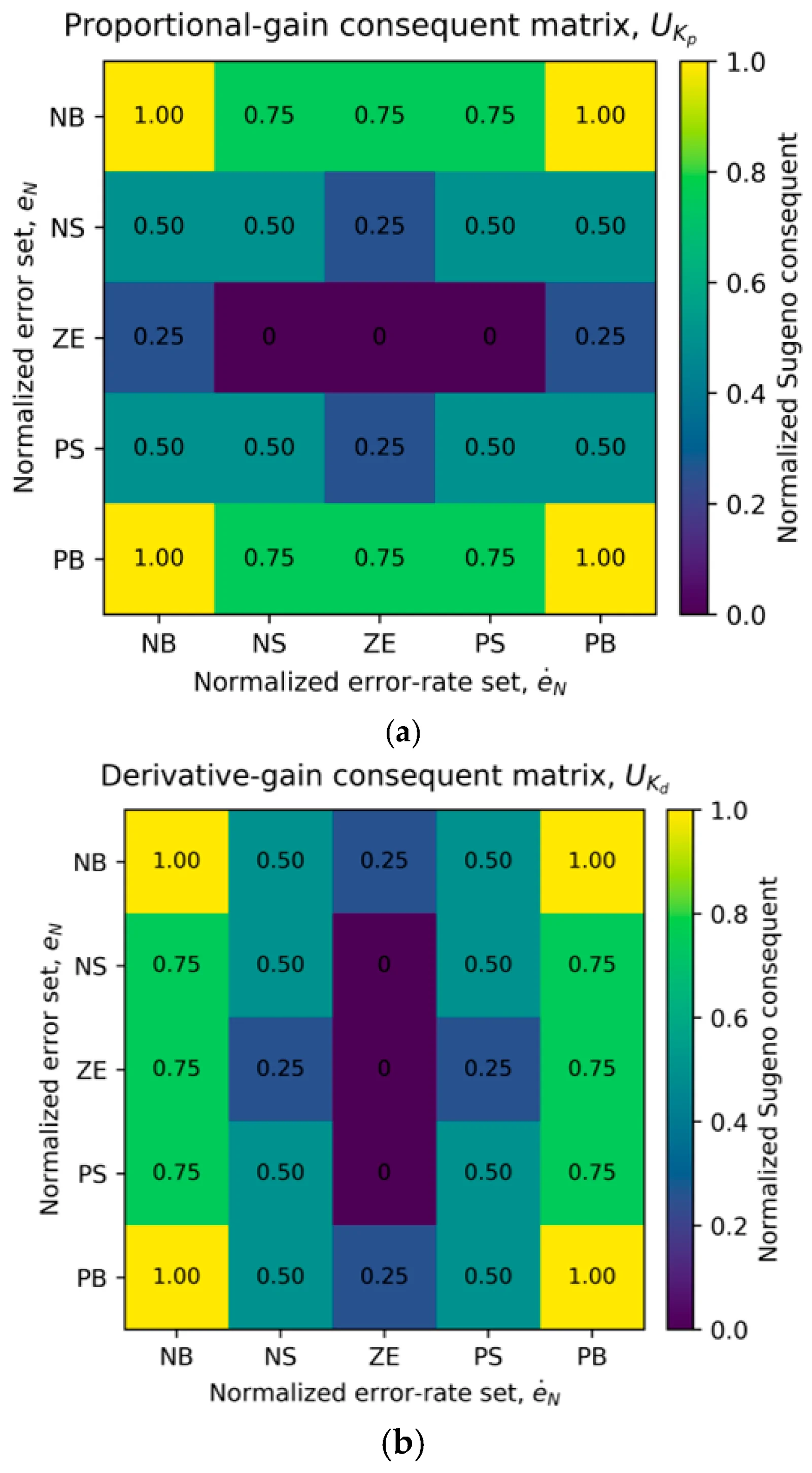
Zero-order Sugeno consequent matrices for (**a**) proportional- and (**b**) derivative-gain supervision.

The proportional matrix emphasizes larger position errors, whereas the derivative matrix emphasizes larger error-rate magnitudes. Both matrices are symmetric with respect to sign; therefore, the supervisor does not encode a preferred direction of motion. The proportional- and derivative-gain consequent matrices are illustrated in Figure 6.

The physical controller gains are reconstructed as:

(18) $$K_{p,i}\left(t\right)=K_{p0}+\Delta K_{p,max}u_{Kp,i}\left(t\right)$$

(19) $$K_{d,i}\left(t\right)=K_{d0}+\Delta K_{d,max}u_{Kd,i}\left(t\right)$$

with *ΔK_p,max_* = 4 N/mm and *ΔK_d,max_* = 0.165 N·s/mm. Hence, *K_p_*(*t*) is restricted to [20, 24] N/mm and *K_d_*(*t*) to [0.5, 0.665] N·s/mm, corresponding to maximum increases of 20% and 33%, respectively. No GA, PSO, or other formal optimization was used. These bounds were selected conservatively and fixed before the reported comparison set; they should not be interpreted as globally optimal gains.

The FSPID feedback contribution is:

(20) $$F_{FSPID,i}=K_{p,i}e_{i}+K_{i}z_{i}+K_{d,i}\dot{e_{i}}$$

As for the fixed-gain PID, the total FSPID actuator command is obtained by adding the common gravity-compensation term to the feedback contribution and then applying the common diagnostic limiter. Table 3 summarizes the controller and fuzzy-supervisor parameters used in all reported comparisons.

The parameters in Table 3 were not retuned between scenarios. This matched-parameter protocol is used so that differences between PID and FSPID can be attributed to the bounded supervisory action rather than to scenario-specific tuning.

### 2.5. Simulation Protocol and Robustness Scenarios

All final comparisons were performed using the same CAD-derived Simscape Multibody plant, with the controller parameters listed in Table 3, and matched initial conditions for PID and FSPID. Dynamic outputs were evaluated on a common 0.002 s grid, irrespective of the internal variable-step solver time points, to prevent differences in solver sampling from affecting the calculated performance indicators.

The nominal tracking case used the 1.0° Rotation.y CAD-derived trajectory at 0.2 Hz for 5 s. Three sustained external-load tests used zero actuator reference for 30 s while applying either *Mz* = +1 N·m, *Fy* = +10 N, or *Fz* = +10 N through the CAD-defined moving-platform wrench interface. These force and moment values are synthetic disturbance cases selected for comparative simulation and are not claimed to represent measured patient loads.

Sensitivity to synthetic measurement uncertainty was evaluated by adding zero-mean Gaussian perturbations to the error signals supplied to the controllers, while tracking performance was calculated against the clean CAD reference. The base perturbation standard deviations were 0.01 mm for position and 0.10 mm/s for velocity; additional cases used 0.5× and 2× these values. Random seed 2026 was used, and exactly the same noise realization was supplied to PID and FSPID in each matched comparison. These levels are numerical perturbations rather than manufacturer sensor specifications.

Joint-resistance sensitivity was evaluated through the native viscous-damping coefficient of the three prismatic joints. The same coefficient was assigned to all three joints and swept over *b* = 0, 70, 140, and 280 N·s/m. At the maximum nominal actuator speed of approximately 3.571 mm/s, these coefficients correspond to velocity-dependent resisting forces of approximately 0, 0.25, 0.50, and 1.00 N. Because actuator friction was not experimentally identified, this sweep is interpreted as parametric viscous-damping uncertainty rather than as a measured friction model.

A combined perturbation case applied the base synthetic measurement noise together with b = 140 N·s/m during the nominal 1.0° CAD trajectory. Trajectory-demand sensitivity was examined separately using CAD-derived Rotation.y amplitudes of 0.5°, 1.0°, and 1.5° at the same 0.2 Hz frequency. The complete protocol is summarized in Table 4.

For the base 1× noise level, the position and velocity standard deviations are 0.01 mm and 0.10 mm/s, respectively; the 0.5× and 2× cases scale both values by the same factor. All simulations start from the verified neutral CAD configuration with zero actuator velocity and zero leaky-integrator state.

The protocol is entirely simulation-based; no actuator, sensor, patient, communication, or hardware-in-the-loop system was used.

### 2.6. Performance Indicators

Tracking and actuator-force metrics were calculated independently for the three active prismatic joints on the common 0.002 s evaluation grid. For actuator *i* with *N* evaluation samples, the root-mean-square tracking error is:

(21) $${RMSE}_{i}={\left[\frac{1}{N}\sum_{k=1}^{N}{e_{i,k}}^{2}\right]}^{\frac{1}{2}}$$

the maximum absolute tracking error is

(22) $$e_{max,i}={max}_{k}\left|e_{i,k}\right|$$

and the integral of absolute error is

(23) $${IAE}_{i}=\int_{0}^{T}\left|e_{i}\left(t\right)\right|dt$$

Actuator-force demand is characterized by the following peak absolute commanded force:

(24) $$F_{pk,i}={max}_{k}\left|F_{cmd,i,k}\right|$$

and the root-mean-square commanded force

(25) $$F_{RMS,i}={\left[\frac{1}{N}\sum_{k=1}^{N}{F_{cmd,i,k}}^{2}\right]}^{\frac{1}{2}}$$

Mean tracking indicators are obtained by averaging the corresponding actuator-wise metrics over the three active joints. The global peak actuator force is the largest *F_pk,i_* among the three joints. Force metrics are computed from the total commanded prismatic-joint force, including the common gravity-compensation term. Percentage comparisons are calculated actuator by actuator and then averaged. For an actuator-wise metric X with a nonzero PID baseline value, the mean percentage change is:

(26) $$\Delta X(\%)=\frac{1}{3}\sum_{i=1}^{3}100\frac{X_{FSPID,i}-X_{PID,i}}{X_{PID,i}}$$

With this sign convention, a negative percentage indicates a lower metric for FSPID than for PID, whereas a positive value indicates an increase. For tracking-error metrics, negative values therefore represent an improvement. For force metrics, the sign is reported without interpreting a force increase as beneficial or detrimental in isolation.

In addition to the performance indicators, the FSPID implementation was audited for normalized-input clipping, effective *K_p_*(*t*) and *K_d_*(*t*) ranges, and diagnostic force-command saturation. These checks are reported as numerical operating-range evidence and do not replace hardware safety validation.

## 3. Results

Results are reported on the common 0.002 s evaluation grid defined in Section 2.6. Negative signed changes in RMSE, maximum absolute error, and IAE indicate lower FSPID tracking error; actuator-force changes are reported algebraically without assigning a beneficial sign. The principal matched comparisons are summarized in Table 5.

### 3.1. Nominal CAD-Derived Tracking Response

**Figure 7:**
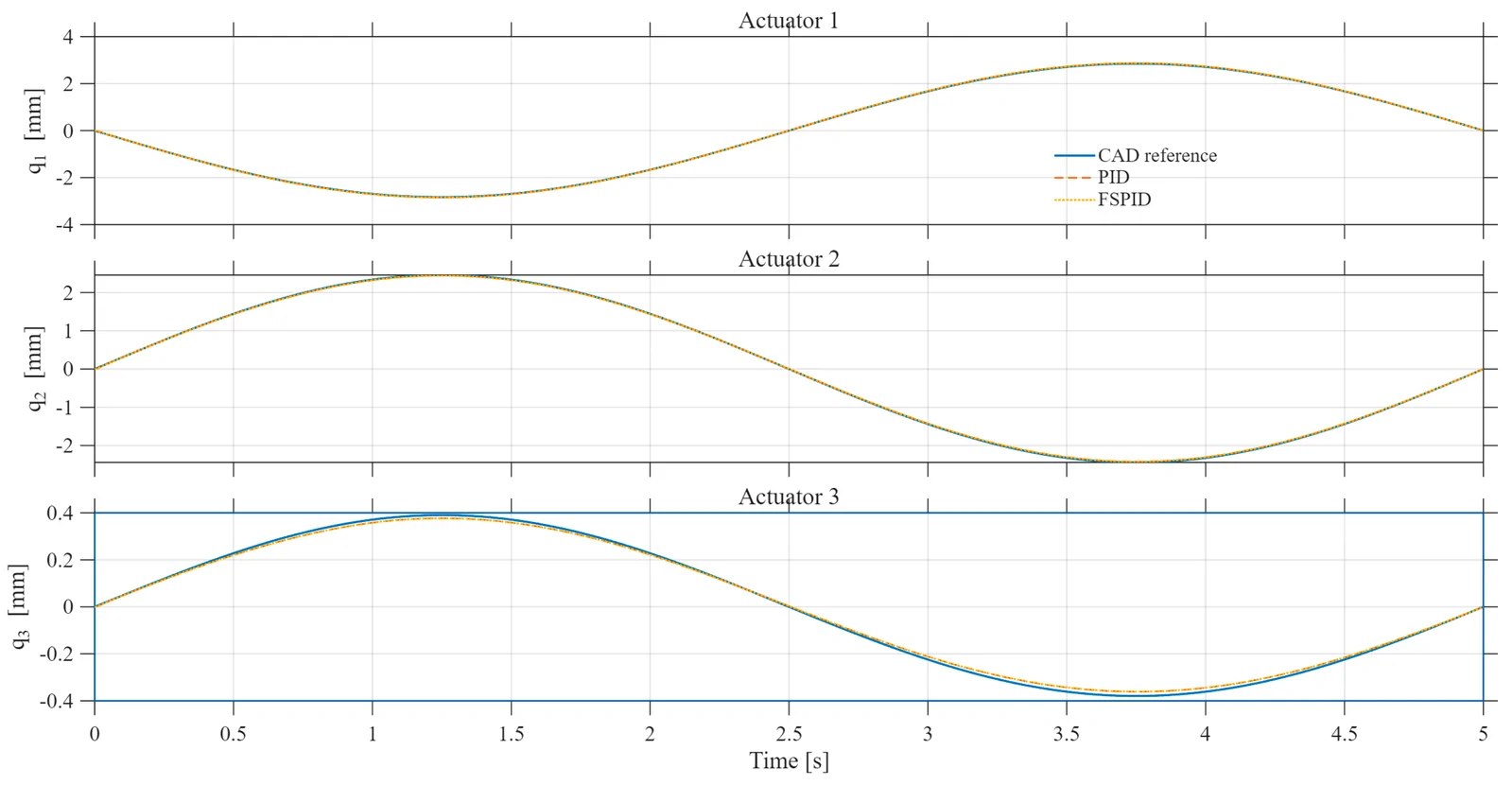
Nominal tracking response for the CAD-derived 1.0° Rotation.y trajectory at 0.2 Hz.

The nominal comparison used the CAD-derived 1.0° Rotation.y trajectory at 0.2 Hz. Both controllers tracked the three prismatic-joint references with small errors. Averaged over the three actuators, PID and FSPID produced RMSE values of 0.011937 and 0.011908 mm, respectively, corresponding to a 0.25% reduction with FSPID. The mean maximum absolute error decreased from 0.019292 to 0.019220 mm (−0.37%), while mean IAE decreased by 0.21%. The global peak commanded force was essentially unchanged at 18.489 N for PID and 18.488 N for FSPID. The corresponding nominal tracking responses are shown in Figure 7.

The small nominal differences are consistent with the conservative supervisory design. The fixed-gain PID already exhibits small tracking error for the low-amplitude nominal trajectory, leaving limited scope for additional proportional and derivative action. The nominal case therefore does not indicate a large FSPID advantage over this baseline.

### 3.2. Response to External Force and Moment Disturbances

**Figure 8:**
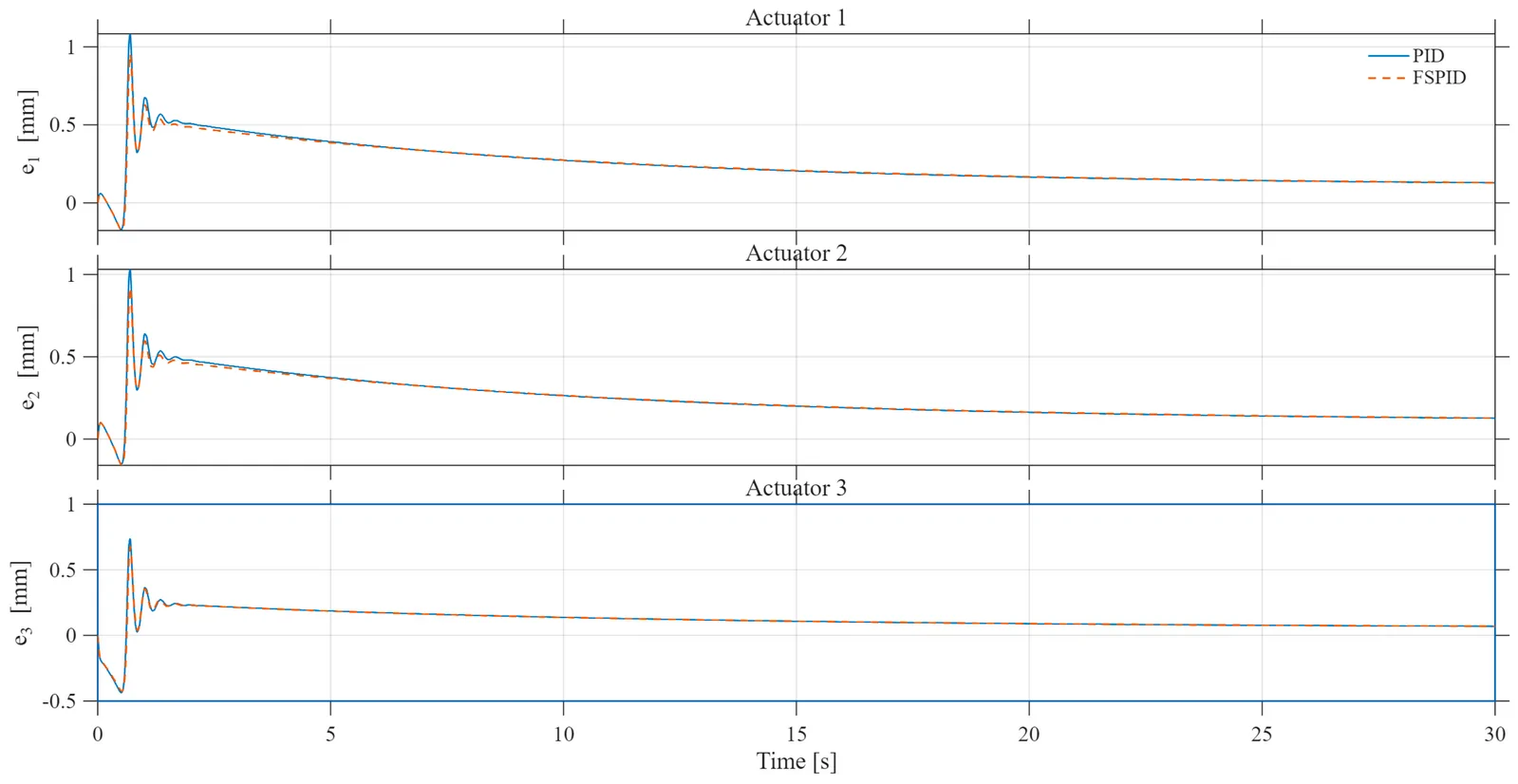
Tracking-error histories for the three active prismatic joints under the sustained Mz = +1 N·m external-moment disturbance.

The distinction between the controllers became more visible under sustained external loading. With *Mz* = +1 N·m applied at the moving-platform load frame for 30 s, FSPID reduced the mean RMSE from 0.22668 to 0.22293 mm (−1.56%) and the mean maximum absolute error from 0.95001 to 0.85062 mm (−10.20%). Mean IAE decreased by 0.60%. The global peak actuator force increased from 39.090 to 39.625 N; the mean actuator-wise peak-force change was +1.05%, and the mean RMS-force change was +0.26%. No diagnostic ±100 N command saturation occurred. The tracking-error histories of the three active prismatic joints under the sustained external-moment disturbance are shown in Figure 8.

For the synthetic *Fy* = +10 N case, FSPID reduced the mean RMSE by 1.29%, the mean maximum absolute error by 9.33%, and the mean IAE by 0.55%. The global peak force decreased from 22.667 to 22.045 N, while the mean actuator-wise peak-force change was −0.91%. For the synthetic *Fz* = +10 N case, the corresponding tracking changes were −3.15% in RMSE, −10.07% in maximum absolute error, and −0.79% in IAE. In this case, the global peak force increased from 47.275 to 48.119 N, and the mean actuator-wise peak-force change was +1.83%. Thus, the largest FSPID differences in the core protocol were observed in maximum tracking error during sustained external loading, whereas the associated actuator-force changes remained comparatively small.

### 3.3. Sensitivity to Synthetic Measurement Noise and Viscous Joint Damping

The synthetic measurement-noise study showed a non-monotonic relative difference between the controllers. At 0.5× the base noise level, the mean changes in RMSE, maximum absolute error, and IAE were −0.26%, −0.36%, and −0.29%, respectively. At the base 1× level (*σ_q_* = 0.01 mm and *σ_v_* = 0.10 mm/s), the corresponding changes were −0.50%, −0.46%, and −0.53%. At 2× noise, the mean RMSE change was +0.10% and the maximum-error change was +1.87%, although IAE remained nearly unchanged (−0.04%). FSPID therefore did not provide a uniform tracking advantage at the highest investigated synthetic-noise level.

Viscous-joint-damping sensitivity was examined using *b* = 0, 70, 140, and 280 N·s/m for all three prismatic joints. FSPID retained lower mean RMSE and IAE at every tested damping value. The maximum-error metric was also lower except at *b* = 70 N·s/m, where the signed change was +1.03%. Peak- and RMS-force changes remained below 0.01% in magnitude throughout this sweep. Because these coefficients were not experimentally identified, the results quantify sensitivity to a model parameter rather than robustness to measured actuator friction.

**Figure 9:**
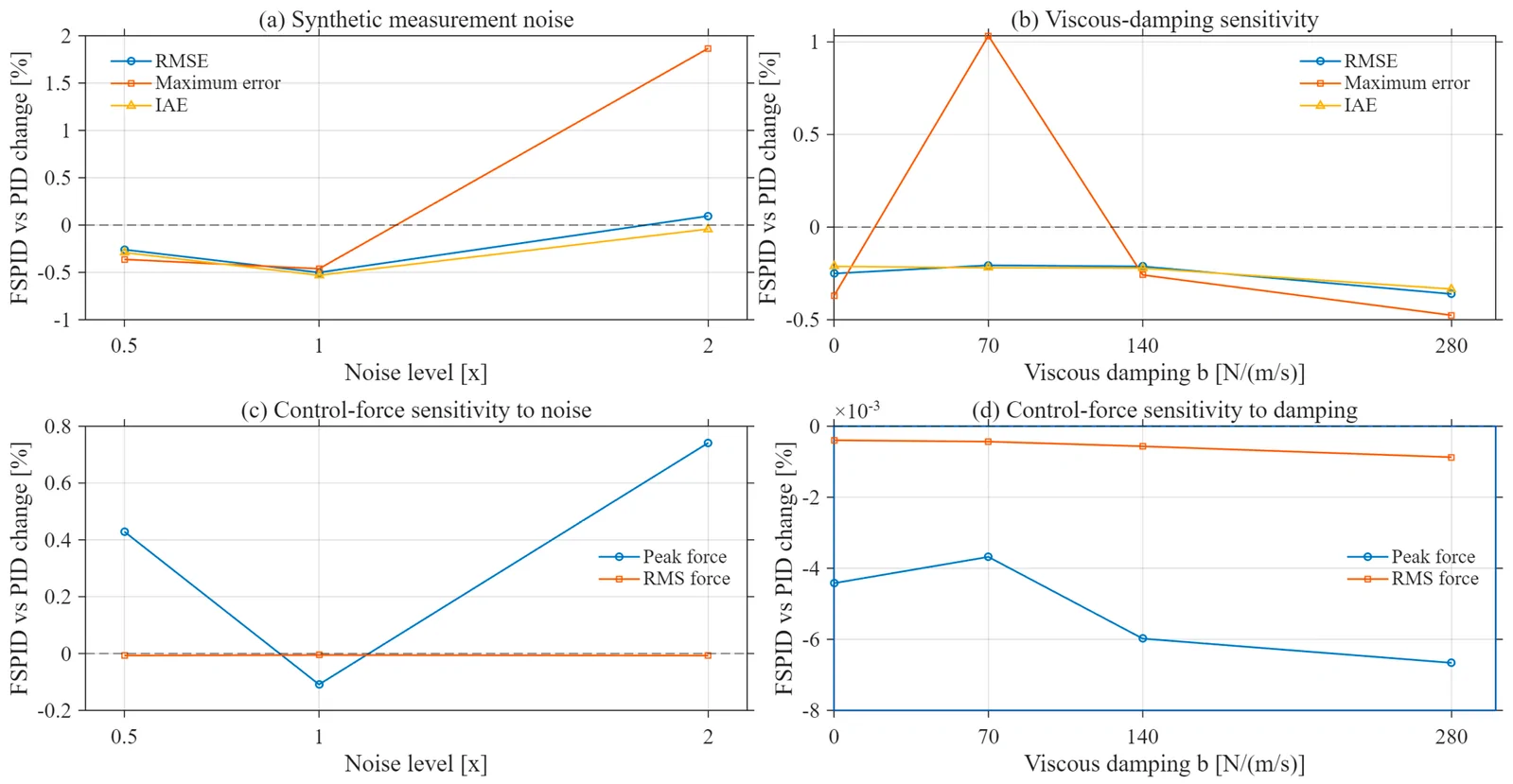
Robustness and sensitivity comparison between PID and FSPID: (**a**) relative changes in RMSE, maximum tracking error, and IAE under different synthetic measurement-noise levels; (**b**) relative changes in RMSE, maximum tracking error, and IAE for different parametric viscous-joint-damping values; (**c**) relative changes in peak and RMS commanded actuator force under different synthetic measurement-noise levels; and (**d**) relative changes in peak and RMS commanded actuator force for different parametric viscous-joint-damping values.

The comparative results of the synthetic measurement-noise and viscous-joint-damping on both tracking performance and actuator-force demand are summarized in Figure 9.

When the base 1× synthetic noise was combined with *b* = 140 N·s/m, the mean RMSE, maximum absolute error, and IAE changes were −0.37%, −0.41%, and −0.33%, respectively. The mean peak-force and RMS-force changes were −0.02% and approximately 0.00%, and no diagnostic command saturation was observed. Tracking remained numerically stable and bounded in this combined perturbation case, while the difference between FSPID and PID remained modest. The signed relative changes obtained in the noise, damping, combined-perturbation, and trajectory-amplitude sensitivity analyses are summarized in Table 6.

### 3.4. Trajectory-Amplitude Sensitivity

**Figure 10:**
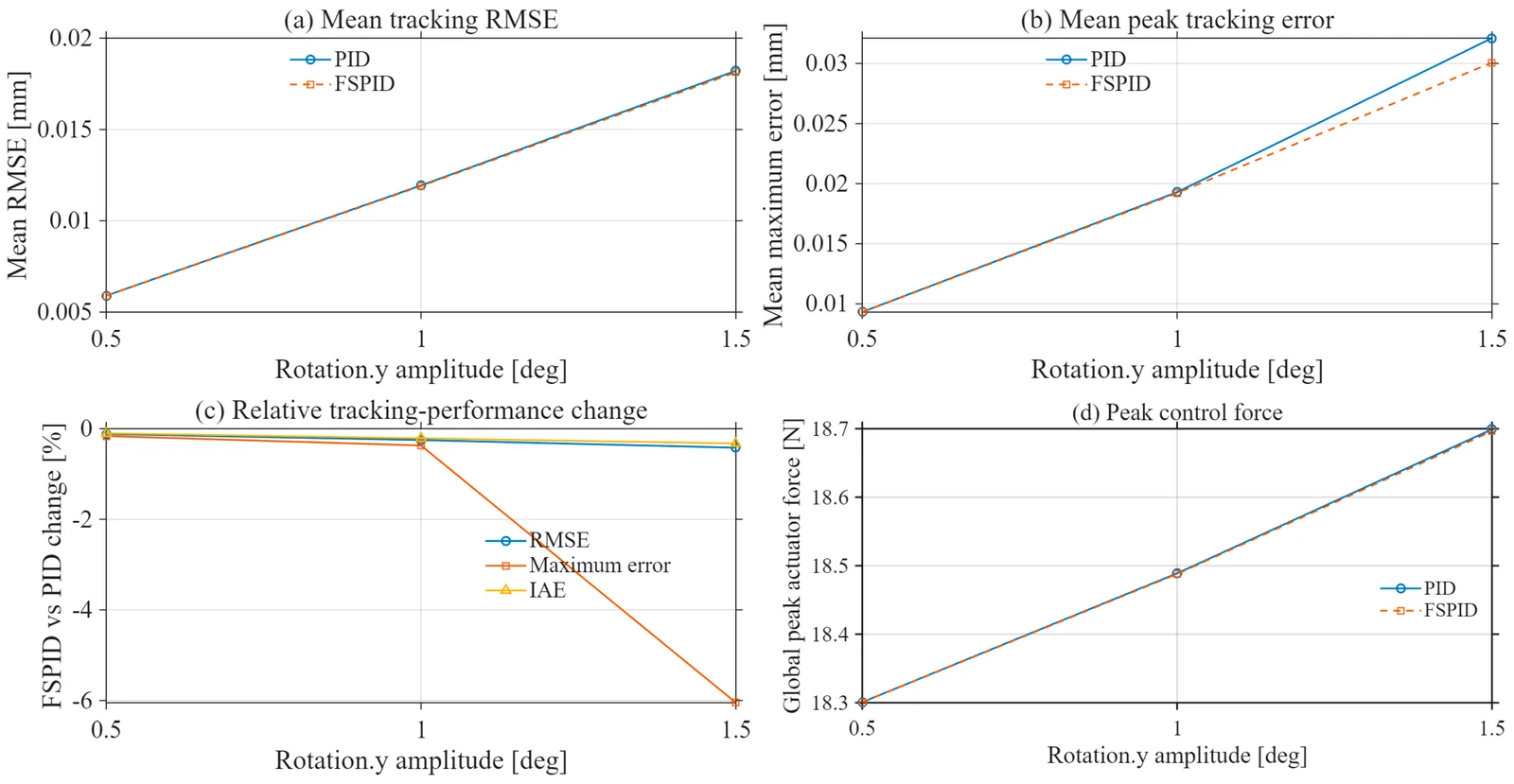
Trajectory-amplitude sensitivity for CAD-derived Rotation.y references at 0.5°, 1.0°, and 1.5°: (**a**) mean tracking RMSE for PID and FSPID; (**b**) mean maximum tracking error for PID and FSPID; (**c**) relative changes in RMSE, maximum tracking error, and IAE for FSPID with respect to PID; and (**d**) global peak commanded actuator force for PID and FSPID.

The CAD-derived inverse-kinematic trajectories at 0.5°, 1.0°, and 1.5° were all solved continuously over 2501 samples before closed-loop evaluation. Mean PID/FSPID RMSE values were 0.0059013/0.0058944 mm at 0.5°, 0.011937/0.011908 mm at 1.0°, and 0.018213/0.018134 mm at 1.5°. The corresponding FSPID RMSE changes were −0.12%, −0.25%, and −0.42%. Mean maximum-error changes were −0.16%, −0.37%, and −6.05%, respectively. Peak actuator force remained essentially unchanged across the amplitude sweep, and no diagnostic saturation was observed. The effects of trajectory amplitude on the tracking-error metrics, relative FSPID performance, and peak actuator-force demand are summarized in Figure 10.

Within the investigated amplitude range, the relative reduction in maximum tracking error was largest for the 1.5° trajectory. This is a local sensitivity observation and does not establish performance over the full mechanism workspace or near singular configurations.

### 3.5. Fuzzy-Supervisor Operating Range and Diagnostic Limits

The fuzzy supervisor remained within its prescribed operating range in the audited sustained-load case. During the 30 s with *Mz* = +1 N·m simulation, the maximum absolute normalized fuzzy input remained below 0.8, so clipping at ±1 was not activated. The maximum observed proportional gains for actuators 1–3 were 22.91, 22.80, and 22.21 N/mm, respectively, below the 24 N/mm limit. The corresponding maximum derivative gains were 0.636, 0.634, and 0.627 N·s/mm, below the 0.665 N·s/mm limit. The supervisor therefore remained below its prescribed gain limits and did not continuously reach either maximum allowable gain.

No saturation of the diagnostic ±100 N bound (defined in Section 2.3) occurred in the core comparison set, the combined noise–damping case, or the trajectory-amplitude sweep. This observation must not be interpreted as validation of actuator capacity or human-interaction safety, because neither a physical actuator specification nor an experimentally established clinical force limit is represented by this bound.

## 4. Discussion

The results show that FSPID acts as a conservative supervisory refinement of fixed-gain PID within the investigated CAD-derived multibody model. Nominal differences are small, whereas reductions in maximum tracking error are more visible under sustained external loading and at the largest investigated trajectory amplitude. These findings should be interpreted as mechanism-level simulation results rather than as evidence of clinical or experimental performance.

### 4.1. Influence of the CAD-Derived Multibody Plant

Using Simscape Multibody avoids reliance on a constant pairwise actuator-coupling representation for the closed-loop 3-DOF mechanism. The mass matrix, constraint Jacobian, gravitational terms, and load transmission vary with the instantaneous multibody configuration. This distinction is relevant for parallel rehabilitation mechanisms, for which geometry, Jacobian properties, workspace, and load transmission are central design considerations [7,8,9]. The controller comparison is therefore performed against mechanism-level rigid-body dynamics rather than an imposed symmetric coupling matrix.

The mechanism-level model remains distinct from an experimentally identified electromechanical prototype. Motor electrical dynamics, transmission compliance and backlash, measured linear-guide friction, and structural flexibility are not available from the current CAD model. The viscous-damping study therefore represents parameter sensitivity rather than friction identification, and the inverse-/forward-dynamics consistency check demonstrates internal numerical agreement between two simulation formulations rather than experimental validation. Prototype-oriented rehabilitation studies show that kinematic and control verification must ultimately be complemented by physical-system characterization and laboratory testing [6,8]. Measured actuator and joint characteristics could alter both the baseline tuning and the relative benefit of fuzzy supervision.

### 4.2. Interpretation of the Fuzzy Supervision and Leaky Integral Action

The fuzzy layer adjusts only *K_p_* and *K_d_* within narrow one-sided bounds. No formal optimization algorithm, such as GA or PSO, was used to select the +20% proportional and +33% derivative limits; they were chosen as conservative engineering bounds after establishing the fixed-gain baseline and were then frozen for the reported comparisons. This differs from rehabilitation-robot studies in which PID gains are optimized offline [5] or fuzzy/adaptive layers modify assistance more broadly [10,11,12,13,14,15,16,17,18].

Table 7 situates the present gain-adaptation approach against five recently published fuzzy or fuzzy-adaptive controllers for rehabilitation robots, summarizing the reported fuzzy input variables, the adapted parameter and its range where stated, the test conditions, and the quantitative improvement each study reported. The comparison shows considerable heterogeneity in mechanism type, adaptation target, and evaluation metric: reported outcomes range from a qualitative demonstration [10] to an approximately five-fold tighter simulated tracking-error bound [11], a training-versus-retention trade-off with no uniform winner [12], and RMSE reductions of roughly 29–33% under simulation-only testing [13]; one study’s accessible text did not report a quantitative baseline comparison [14]. The present supervisor adapts a comparatively narrow, one-sided range (+20% on *K_p_*, +33% on *K_d_*) around a fixed baseline and reports mean maximum-tracking-error reductions of 9.33–10.20% under sustained external loading and 0.25–0.37% under nominal CAD-derived tracking (Table 5). Given the differing mechanisms, metrics, and experimental conditions across these studies, this comparison is presented as a qualitative positioning of the design choice rather than a numerical ranking.

The simulated gain histories remained below the prescribed limits even during the sustained-moment test, consistent with bounded supervisory correction rather than unrestricted online retuning.

Keeping *K_i_* fixed is also deliberate. During sustained-load numerical checks, a pure integral state produced progressive internal-force accumulation associated with a weak actuator-space direction of the closed-chain mechanism. Reducing *K_i_* slowed this accumulation but did not eliminate the underlying long-memory behavior. The final controller therefore retains *K_i_* = 2 N/(mm·s) while replacing the pure integrator with a leaky state using *λ* = 0.02 s^−1^. This value was selected as a practical trade-off from a small sensitivity study and is not claimed to be globally optimal. Adapting *K_i_* could improve steady-state rejection in some systems, but—as explained in Section 2.3—it would risk introducing a second slow supervisory channel affecting internal preload under sustained loading. More generally, interaction-control literature emphasizes that controller behavior under contact depends on the coupled robot–environment dynamics rather than on position error alone [18,19,20,21,22,23].

### 4.3. External Loading, Noise, and Joint-Resistance Sensitivity

The explicit external-load cases extend the nominal trajectory tests by introducing sustained force and moment disturbances. Under the 1 N·m moment and two 10 N force cases, FSPID reduced the mean maximum tracking error by approximately 9.3–10.2%. The corresponding RMSE and IAE changes were smaller, indicating that the supervisory action primarily reduced the largest deviations rather than producing a large uniform reduction over the complete 30 s interval. Actuator-force changes were case-dependent and comparatively small. This disturbance-oriented evaluation is consistent with the physical-interaction perspective emphasized in the rehabilitation and pHRI control literature [10,18,19,21,22,23].

These external loads are synthetic engineering disturbances. They do not reproduce patient limb impedance, voluntary effort, fatigue, pain, or spasticity, and the CAD-defined load application frame is not a validated anatomical interface. Therefore, the results demonstrate disturbance-rejection behavior of the simulated mechanism only. A patient-coupled model or instrumented physical interface would be required before drawing conclusions about rehabilitation interaction performance. Impedance, admittance, and patient-cooperative approaches provide established frameworks for such future interaction-level control and evaluation [18,19,21,22,23].

The noise and damping studies support only a limited robustness interpretation. FSPID produced modest error reductions at the 0.5× and 1× synthetic noise levels, but the 2× case did not show uniform superiority in RMSE and maximum error. This loss of consistent advantage is relevant because derivative-dependent supervision is sensitive to measurement quality. The viscous-damping sweep showed small differences between controllers and one isolated increase in maximum error at *b* = 70 N·s/m. The results therefore do not support a general claim of noise or friction robustness; they document numerical sensitivity over explicitly defined perturbation ranges. This interpretation is consistent with the pHRI literature emphasizing the roles of sensing, filtering, apparent inertia, damping, and coupled interaction dynamics [22,23].

### 4.4. Rehabilitation Relevance, Fault Tolerance, and Cybersecurity Considerations

Accurate prismatic-joint tracking is relevant to repeatable orientation control of the moving platform, but trajectory accuracy is not a clinical outcome. The robot-assisted rehabilitation literature spans position-controlled, patient-cooperative, impedance/admittance, adaptive, assist-as-needed, and socially assistive approaches, with clinical benefit depending on the complete therapeutic protocol rather than on actuator tracking alone [1,2,3,4,16,17,18,19,20,21,22,23,24,25,26,27,28,29]. The present simulations do not quantify therapeutic effectiveness, comfort, patient safety, or clinical benefit. For active rehabilitation, an actuator-position layer such as the one studied here would normally be embedded within a higher-level interaction controller such as, for example, impedance, admittance, force-position, or assist-as-needed control, with experimentally established human–robot limits [10,12,18,19,21,22,23]. Functional validation studies of parallel rehabilitation devices further illustrate the need for prototype-level safety limits and laboratory testing before human use [6,15].

The three controller loops are decentralized at the feedback-law level and rely on the common mechanical plant for coordination. This architecture does not provide a formal consensus or fault-tolerance guarantee if an actuator, sensor, or communication link fails. Multi-agent dynamic-surface consensus methods developed for other safety-critical multi-actuator systems explicitly address synchronization under communication faults [30]. Although the steer-by-wire application in [30] is not a rehabilitation system, it provides relevant methodological context for a future extension in which actuator agreement, fault detection, and degraded-mode coordination are treated explicitly rather than being left to passive mechanical coupling.

Networked rehabilitation platforms introduce risks that are outside the present simulation model, including command or sensor spoofing, unauthorized access, packet delay, and loss of data integrity. A future connected implementation should separate safety-critical local control from non-critical network services, authenticate devices and commands, protect data in transit, monitor anomalous behavior, and apply least-privilege or zero-trust access principles. General zero-trust guidance removes implicit trust based solely on network location [26], while the dynamic zero-trust framework in [31] provides a complementary risk-assessment perspective. Medical-device cybersecurity guidance likewise emphasizes cybersecurity-by-design and lifecycle risk management [27]. In a different safety-critical electromechanical domain, Ref. [32] illustrates how sensing can be combined with physics-based constraints for state-consistency monitoring; an analogous principle could be investigated for future rehabilitation-platform monitoring.

As noted in Section 2.3, the ±100 N command limiter used in this study is a numerical diagnostic bound only, not a validated physical safety threshold. A prototype would require experimentally established actuator current/force limits, joint and workspace limits, independent emergency stopping, watchdogs, fault detection, and interaction-force supervision. For a medical-device development path, these controls would need to be incorporated into a formal risk-management and software-lifecycle process rather than inferred from a simulation saturation value [24,25]. Secure communication and cybersecurity controls [26,27,31] cannot replace local physical safety mechanisms; cyber protection and fail-safe design must be treated as complementary layers.

### 4.5. Limitations and Experimental Validation Path

The main limitation of this study is the absence of hardware validation. No physical prototype, hardware-in-the-loop platform, measured sensor-noise spectrum, experimentally identified friction model, patient-interaction dataset, or embedded execution-time benchmark was available. Accordingly, the study does not demonstrate real-time feasibility, actuator thermal capability, clinical safety, or human-interaction performance. The zero-order Sugeno supervisor contains only two inputs and 25 rules and is algorithmically compact, but computational simplicity should not be confused with measured real-time performance on a target processor. Any future medical-device implementation would also require structured risk management, software lifecycle controls, and cybersecurity engineering beyond the controller algorithm itself [24,25,26,27].

A logical next validation sequence is: first, identify actuator and transmission dynamics on a physical mechanism; second, establish mechanical workspace, force, current, and emergency-stop limits; third, compare PID and FSPID in hardware-in-the-loop and benchtop experiments under measured disturbances and sensor noise; and only then consider supervised human-interaction studies under an approved protocol. This staged progression is consistent with the distinction between simulation studies and prototype/clinical validation reported in the rehabilitation-robot literature [6,15,16,17,18,19,20,28]. Fault injection for actuator/sensor/communication failures and timing measurements on the intended embedded controller should be included before any claim of real-time or safety-critical readiness, together with the risk, software, and cybersecurity processes required for connected medical systems [24,25,26,27].

## 5. Conclusions

This study evaluated a bounded zero-order Sugeno fuzzy-supervised PID controller for a 3STC+S parallel rehabilitation mechanism using a CAD-derived Simscape Multibody model. CAD-consistent inverse kinematics generated the three prismatic-joint references, while the forward-dynamics plant retained rigid-body mass and inertia properties, gravity, closed-loop constraints, and an external force/moment interface. FSPID adjusted only *K_p_* and *K_d_* within prescribed bounds and used the same fixed leaky integral action and neutral gravity compensation as the PID baseline.

Under the nominal 1.0° trajectory, FSPID produced only small mean reductions in RMSE, maximum absolute error, and IAE of 0.25%, 0.37%, and 0.21%, respectively, with essentially unchanged peak actuator force. Under sustained external loading, the difference was more pronounced in maximum tracking error: reductions of 10.20%, 9.33%, and 10.07% were obtained for the *Mz* = +1 N·m, *Fy* = +10 N, and *Fz* = +10 N cases, respectively. The amplitude study showed a larger relative reduction in maximum tracking error at the investigated 1.5° trajectory, whereas the highest synthetic-noise level did not produce uniform FSPID superiority. No diagnostic force-command saturation occurred in the principal reported comparisons.

The results support FSPID as a conservative simulation-level refinement of fixed-gain PID for the investigated CAD-derived multibody model, particularly under increased transient demand or sustained external loading. These findings do not establish clinical benefit, experimental robustness, real-time feasibility, or safety. Physical actuator identification, hardware testing, measured human–robot interaction, fault-tolerant coordination, embedded timing evaluation, and cyber–physical safety validation remain necessary before practical rehabilitation use.

## Figures and Tables

**Table 1:** CAD-derived inverse-kinematic reference-generation settings.

Parameter	Value
Multibody environment	Simscape Multibody
Reference-generation method	Closed-loop inverse kinematics
Active coordinates	Three prismatic-joint displacements
Driven platform coordinate	Rotation.y
Nominal angular amplitude	1.0°
Amplitude-sensitivity cases	0.5°, 1.0°, 1.5°
Excitation frequency	0.2 Hz
Sampling interval, Ts	0.002 s
Reference duration	5 s
Samples per trajectory	2501
Initial configuration	Verified neutral CAD configuration
Branch-continuity strategy	Previous valid solution used to initialize the subsequent sample

**Table 2:** CAD-derived rigid-body and multibody-model settings.

Item	Value/Implementation
Actuator body	3 instances; 0.774845 kg per instance; CAD-derived inertia retained
Actuator rod	3 instances; 0.181229 kg per instance; CAD-derived inertia retained
Universal Joint	3 instances; 0.000994 kg per instance; CAD-derived inertia retained
Fixed platform	1 instance; 0.741335 kg; CAD-derived inertia retained
Moving platform	1 instance; 0.708579 kg; CAD-derived inertia retained
Active joints	3 force-actuated prismatic joints
Passive/constraint joints	6 revolute joints and 4 spherical joints; closed loops solved as multibody constraints
Gravity vector	[0, −9.80665, 0] m/s^2^
Nominal prismatic viscous damping	0 N·s/m
External-load interface	Six-component force/moment input at a CAD-defined moving-platform frame
Common evaluation interval	0.002 s

**Table 3:** Final fixed PID and fuzzy-supervisor parameters used in the simulation study.

Parameter	Value
Baseline proportional gain, *K_p0_*	20 N/mm
Integral gain, *K_i_*	2 N/(mm·s)
Baseline derivative gain, *K_d0_*	0.5 N·s/mm
Leaky-integrator parameter, *λ*	0.02 s^−1^
Error normalization, *Es*	1.3 mm
Error-rate normalization, *Eds*	16.3 mm/s
Normalized input interval	[−1, 1]
Maximum proportional increment, *ΔK_p,max_*	4 N/mm (+20%)
Maximum derivative increment, *ΔK_d,max_*	0.165 N·s/mm (+33%)
Admissible *K_p_*(*t*)	[20, 24] N/mm
Admissible *K_d_*(*t*)	[0.5, 0.665] N·s/mm
Fuzzy sets per input	5 (NB, NS, ZE, PS, PB)
Number of rules	25
Inference type	Zero-order Sugeno
Conjunction operator	Product
Output constants	0, 0.25, 0.50, 0.75, 1.00
Integral adaptation	None; *K_i_* and *λ* fixed
Common diagnostic command bound	±100 N
Neutral gravity-compensation vector, *F_g_*	[15.221, −13.171, −18.127]^T^ N

**Table 4:** Final matched PID–FSPID simulation protocol.

Scenario	Simulation Condition	Duration
Nominal CAD tracking	Rotation.y = 1.0°, 0.2 Hz; no added load/noise/damping	5 s
External moment	Zero actuator reference; *Mz* = +1 N·m	30 s
External force *Fy*	Zero actuator reference; *Fy* = +10 N	30 s
External force *Fz*	Zero actuator reference; *Fz* = +10 N	30 s
Synthetic-noise sweep	Nominal CAD trajectory; 0.5×, 1×, and 2× synthetic noise	5 s/case
Viscous-damping sweep	Nominal CAD trajectory; *b* = 0, 70, 140, 280 N·s/m	5 s/case
Combined nonidealities	Nominal CAD trajectory; 1× noise + b = 140 N·s/m	5 s
Amplitude sensitivity	Rotation.y = 0.5°, 1.0°, 1.5° at 0.2 Hz	5 s/case

**Table 5:** Core PID–FSPID tracking results obtained with the CAD-derived Simscape Multibody plant. Tracking metrics are averaged over the three active prismatic joints; global peak force is the largest absolute commanded force among the three joints. Percentage changes use the sign convention of Section 2.6.

Scenario	Mean RMSE PID [mm]	Mean RMSE FSPID [mm]	ΔRMSE [%]	Mean Max. Error PID [mm]	Mean Max. Error FSPID [mm]	*Δmax*. [%]	ΔIAE [%]	Global Peak Force PID/FSPID [N]
Nominal 1°	0.011937	0.011908	−0.25	0.019292	0.019220	−0.37	−0.21	18.489/18.488
*Mz* = +1 N·m	0.22668	0.22293	−1.56	0.95001	0.85062	−10.20	−0.60	39.090/39.625
*Fy* = +10 N	0.18847	0.18445	−1.29	0.51154	0.46058	−9.33	−0.55	22.667/22.045
*Fz* = +10 N	0.27383	0.26582	−3.15	1.1176	0.99867	−10.07	−0.79	47.275/48.119
Noise + damping	0.017833	0.017770	−0.37	0.031077	0.030951	−0.41	−0.33	19.010/18.993

**Table 6:** Signed relative changes for the sensitivity studies. Negative tracking values indicate lower FSPID error. Damping values are parametric model coefficients, and the noise levels are synthetic numerical perturbations.

Sensitivity Case	ΔRMSE [%]	ΔMax. Error [%]	ΔIAE [%]	Mean ΔPeak Force [%]	Mean ΔRMS Force [%]
Noise 0.5×	−0.26	−0.36	−0.29	+0.43	−0.007
Noise 1×	−0.50	−0.46	−0.53	−0.11	−0.005
Noise 2×	+0.10	+1.87	−0.04	+0.74	−0.007
*b* = 70 N·s/m	−0.21	+1.03	−0.22	−0.004	−0.0004
*b* = 140 N·s/m	−0.21	−0.26	−0.22	−0.006	−0.0006
*b* = 280 N·s/m	−0.36	−0.48	−0.33	−0.007	−0.0009
Noise 1× + b = 140 N·s/m	−0.37	−0.41	−0.33	−0.02	−0.0007
Amplitude 0.5°	−0.12	−0.16	−0.10	−0.001	—
Amplitude 1.0°	−0.25	−0.37	−0.21	−0.004	—
Amplitude 1.5°	−0.42	−6.05	−0.33	−0.012	—

**Table 7:** Comparison with related fuzzy/adaptive control schemes for rehabilitation robots.

Study	Mechanism	Fuzzy Input variables	Adapted Parameter (Range)	Test Conditions	Reported Improvement
Ju et al. [10] (2005)	Serial robot guiding wrist along linear/circular trajectories	Not reported in accessible sources	Hybrid position/force control (fuzzy logic); adaptation range not reported	Hardware; human subjects; predefined external force levels	Qualitative demonstration only; no quantitative baseline comparison reported
Zhang et al. [11] (2020)	Lower-limb exoskeleton (2-DOF hip/knee validated)	Output tracking-error vector	Adaptive fuzzy-logic compensation weights; range not reported	Simulation (sinusoidal reference) + hardware interaction test	Tracking error ≈ ±0.01 rad vs. ±0.05 rad for PID (simulation)
Hu et al. [12] (2023)	Bilateral upper-limb end-effector robot, 3-DOF	Task-performance score; force impulse	Assistance stiffness *K* ∈ [0, 12] N/m (adaptive) vs. fixed *K* = 12 N/m	Hardware; 8 healthy subjects; circle-tracing task	No uniform advantage: fixed gain scored higher in training (93.61 vs. 90.63); adaptive scored higher at retention (89.69 vs. 82.93)
Tucan et al. [13] (2025)	4-DOF cable-actuated hand exoskeleton	Tracking error; estimated patient effort	PID gains, fuzzy-modulated; specific gain/range not reported	Simulation only; 3 interaction modes (passive/active-assistive/resistive)	RMSE reduced ≈29–33% vs. fixed PID across modes
Ramirez-Domínguez et al. [14] (2025)	Parallel 4 bar mechanism, 2 actuated joints	Not reported in accessible sources (Takagi–Sugeno/ANFIS plant model)	State-feedback pole placement via Gray Wolf Optimizer; range not reported	Simulation + real-time hardware	Not reported in accessible sources
Present study	3-chain (3STC+S) parallel mechanism, 3 actuated joints	Normalized tracking error; normalized error rate	*K_p_* +20%/*K_d_* +33%, one-sided, bounded around fixed baseline; *K_i_* fixed	CAD-derived multibody simulation; nominal + 3 external-load + noise/damping sweeps	Mean max-error reduction 9.33–10.20% under sustained load; 0.25–0.37% under nominal tracking

## Data Availability

The numerical data supporting the reported simulation results are provided in the Supplementary Materials. Additional MATLAB/Simulink model files are available from the corresponding author upon reasonable request.

## Supplementary Materials

The following supporting information can be downloaded at: https://www.mdpi.com/article/10.3390/bioengineering13091029/s1, Table_S1.pdf, CAD-derived inverse-kinematic reference verification; Table_S2.pdf, CAD rigid-body mass/inertia and transform-mapping audit; Table_S3.pdf, inverse-/forward-dynamics numerical consistency check; Table_S4.pdf, fuzzy-supervisor normalized-input and gain-range audit; Table_S5.pdf, detailed robustness and sensitivity results; Table_S6.pdf, reproducibility-file inventory; and Supporting_MATLAB_Simulink_and_Numerical_Files.zip, containing the supporting MATLAB/Simulink scripts, model files, controller files, and essential numerical result files.

## References

[1] Banyai A.D., Brisan C. (2024). Robotics in Physical Rehabilitation: Systematic Review. Healthcare.

[2] Krebs H.I., Hogan N., Aisen M.L., Volpe B.T. (1998). Robot-Aided Neurorehabilitation. IEEE Trans. Rehabil. Eng..

[3] Veerbeek J.M., Langbroek-Amersfoort A.C., van Wegen E.E.H., Meskers C.G.M., Kwakkel G. (2017). Effects of Robot-Assisted Therapy for the Upper Limb after Stroke: A Systematic Review and Meta-Analysis. Neurorehabilit. Neural Repair.

[4] Rad O.L., Brisan C. (2025). Control Algorithms in Robot-Assisted Rehabilitation: A Systematic Review. Appl. Sci..

[5] Joyo M.K., Raza Y., Ahmed S.F., Billah M.M., Kadir K., Naidu K., Ali A., Mohd Yusof Z. (2019). Optimized Proportional–Integral–Derivative Controller for Upper Limb Rehabilitation Robot. Electronics.

[6] Pisla D., Nadas I., Tucan P., Albert S., Carbone G., Antal T., Banica A., Gherman B. (2021). Development of a Control System and Functional Validation of a Parallel Robot for Lower Limb Rehabilitation. Actuators.

[7] Dong M., Zhou Y., Li J., Rong X., Fan W., Zhou X., Kong Y. (2021). State of the Art in Parallel Ankle Rehabilitation Robot: A Systematic Review. J. Neuroeng. Rehabil..

[8] Li J., Zuo S., Zhang L., Dong M., Zhang Z., Tao C., Ji R. (2020). Mechanical Design and Performance Analysis of a Novel Parallel Robot for Ankle Rehabilitation. J. Mech. Robot..

[9] Todor A., Banyai D.V., Brisan C., Banyai A.D. (2025). Aspects Concerning Parallel Robots Used in Rehabilitation. Bioengineering.

[10] Ju M.-S., Lin C.-C.K., Lin D.-H., Hwang I.-S., Chen S.-M. (2005). A Rehabilitation Robot with Force–Position Hybrid Fuzzy Controller: Hybrid Fuzzy Control of Rehabilitation Robot. IEEE Trans. Neural Syst. Rehabil. Eng..

[11] Zhang X., Li J., Ovur S.E., Chen Z., Li X., Hu Z., Hu Y. (2020). Novel Design and Adaptive Fuzzy Control of a Lower-Limb Elderly Rehabilitation. Electronics.

[12] Hu Y., Meng J., Li G., Zhao D., Feng G., Zuo G., Liu Y., Zhang J., Shi C. (2023). Fuzzy Adaptive Passive Control Strategy Design for Upper-Limb End-Effector Rehabilitation Robot. Sensors.

[13] Tucan P., Vanta O.-M., Vaida C., Ciupe M., Sebeni D., Pisla A., Stiole S., Lupu D., Major Z., Gherman B. (2025). Fuzzy Adaptive Control for a 4-DOF Hand Rehabilitation Robot. Actuators.

[14] Ramirez-Dominguez E.H., Benítez-Morales J.G., Cervantes-Reyes J.E., Alamilla-Daniel M.D.L.A., Licona-Rodríguez A.R., Xicoténcatl-Pérez J.M., Ramos-Fernández J.C. (2025). Identification and Fuzzy Control of the Trajectory of a Parallel Robot: Application to Medical Rehabilitation. Actuators.

[15] Abarca V.E., Elias D.A. (2023). A Review of Parallel Robots: Rehabilitation, Assistance, and Humanoid Applications for Neck, Shoulder, Wrist, Hip, and Ankle Joints. Robotics.

[16] Maciejasz P., Eschweiler J., Gerlach-Hahn K., Jansen-Troy A., Leonhardt S. (2014). A Survey on Robotic Devices for Upper Limb Rehabilitation. J. Neuroeng. Rehabil..

[17] Lo A.C., Guarino P.D., Richards L.G., Haselkorn J.K., Wittenberg G.F., Federman D.G., Ringer R.J., Wagner T.H., Krebs H.I., Volpe B.T. (2010). Robot-Assisted Therapy for Long-Term Upper-Limb Impairment after Stroke. N. Engl. J. Med..

[18] Marchal-Crespo L., Reinkensmeyer D.J. (2009). Review of Control Strategies for Robotic Movement Training after Neurologic Injury. J. Neuroeng. Rehabil..

[19] Riener R., Lunenburger L., Jezernik S., Anderschitz M., Colombo G., Dietz V. (2005). Patient-Cooperative Strategies for Robot-Aided Treadmill Training: First Experimental Results. IEEE Trans. Neural Syst. Rehabil. Eng..

[20] Reinkensmeyer D.J., Emken J.L., Cramer S.C. (2004). Robotics, Motor Learning, and Neurologic Recovery. Annu. Rev. Biomed. Eng..

[21] Hogan N. (1985). Impedance Control: An Approach to Manipulation: Part I-Theory. J. Dyn. Syst. Meas. Control.

[22] Keemink A.Q.L., van der Kooij H., Stienen A.H.A. (2018). Admittance Control for Physical Human-Robot Interaction. Int. J. Robot. Res..

[23] Hogan N. (2022). Contact and Physical Interaction. Annu. Rev. Control Robot. Auton. Syst..

[24] (2019). Medical Devices-Application of Risk Management to Medical Devices.

[25] (2015). Medical Device Software-Software Life Cycle Processes.

[26] Rose S., Borchert O., Mitchell S., Connelly S. (2020). Zero Trust Architecture.

[27] U.S. Food and Drug Administration (2025). Cybersecurity in Medical Devices: Quality Management System Considerations and Content of Premarket Submissions.

[28] Riener R., Nef T., Colombo G. (2005). Robot-Aided Neurorehabilitation of the Upper Extremities. Med. Biol. Eng. Comput..

[29] Mataric M.J., Eriksson J., Feil-Seifer D.J., Winstein C.J. (2007). Socially Assistive Robotics for Post-Stroke Rehabilitation. J. Neuroeng. Rehabil..

[30] Xu K., Liu J., Huang H., Wang C., Zhao W., Ge S.S. (2026). Multi-Agent Dynamic Surface Consensus Control of Dual-Motor Steer-by-Wire System under Communication Faults. IEEE Trans. Ind. Electron..

[31] Wu X., Zou B., Lu C., Wang L., Zhang Y., Wang H. (2025). Dynamic Security Computing Framework With Zero Trust Based on Privacy Domain Prevention and Control Theory. IEEE J. Sel. Areas Commun..

[32] Yan J., Zhou N., Cheng Y., Zhang F., Wang H., Wang M., Jin B., Li M., Lu Q., Zhang W. (2026). Application of Machine-Vision-Driven Physics-Informed Neural Networks in Pantograph-Catenary System State Detection. Mech. Syst. Signal Process..

