# Supplementary material for: Evaluation of a Fuzzy-Supervised PID Controller for a Parallel Rehabilitation Mechanism
Source: Bioengineering (Basel). 2026 Sep 3;13(9):1029. doi: 10.3390/bioengineering13091029 (PMC13603623; doi:10.3390/bioengineering13091029)
Supplement: Supplementary file 1 [file bioengineering-13-01029-s001.zip › Table_S1.pdf]

Table S1. CAD-Derived Inverse-Kinematic Reference Verification

Table S1. CAD-derived inverse-kinematic reference verification.

Quantity	0.5°	1.0°	1.5°	Unit / criterion	Interpretation
Driven platform coordinate	Rotation.y	Rotation.y	Rotation.y	—	Same CAD-defined coordinate for all cases
Excitation frequency	0.2	0.2	0.2	Hz	One period over 5 s
Sampling interval	0.002	0.002	0.002	s	Common reference grid
Reference duration	5	5	5	s	2501 samples including both endpoints
Valid IK samples	2501/2501	2501/2501	2501/2501	samples	No failed inverse-kinematic sample
Maximum actuator-vector increment	0.004738	0.009476	0.014214	mm/sample	No discontinuity indicative of branch switching
Periodic closure error	$< 6 \times 10^{-12}$	$< 6 \times 10^{-12}$	$< 6 \times 10^{-12}$	mm	Numerical closure at machine-precision scale
Branch-continuity strategy	Previous valid solution	Previous valid solution	Previous valid solution	—	Continuation

The nominal 1.0° sample-by-sample actuator reference is provided in A1_CAD_reference.mat. The maximum actuator-vector increment was calculated directly from the stored 2501 × 3 reference array.
