# Supplementary material for: Evaluation of a Fuzzy-Supervised PID Controller for a Parallel Rehabilitation Mechanism
Source: Bioengineering (Basel). 2026 Sep 3;13(9):1029. doi: 10.3390/bioengineering13091029 (PMC13603623; doi:10.3390/bioengineering13091029)
Supplement: Supplementary file 1 [file bioengineering-13-01029-s001.zip › Table_S2.pdf]

Table S2. CAD Rigid-Body Mass/Inertia and Transform-Mapping Audit

Table S2a. CAD-derived rigid-body mass, center-of-mass, and inertia data.

Component	Instances	Mass/instance [kg]	CoM [mm]	MoI [kg·mm²]	PoI [kg·mm²]	Solid index	Mapping
Actuator rod (Tija_Actuator)	3	0.181229120584	[272.887344; -52.245902; 112.024587]	[51.625724; 690.903091; 682.187406]	[20.733063; 129.443702; -105.072572]	1	3/3 OK
Actuator body	3	0.774845129538	[102.437968; -84.833708; 155.572249]	[902.245307; 4609.188967; 4304.099519]	[344.194063; 1161.129174; -841.648883]	2	3/3 OK
Universal Joint	3	0.000993774857	[24.000000; -9.352e-05; -8.010e-05]	[0.042155; 0.023511; 0.023511]	[0; 4.018e-09; -1.930e-09]	3	3/3 OK
Fixed platform	1	0.741335064968	[5.127578; 3.407e-08; 6.589e-09]	[8492.306544; 4255.363726; 4255.363732]	[-9.779e-07; 5.856e-07; -8.618e-08]	4	1/1 OK
Moving platform	1	0.708579424119	[377.410056; -122.335202; 726.207338]	[3481.346455; 8584.044335; 8741.145732]	[-151.509702; 18.682570; -48.402019]	5	1/1 OK

Table S2b. Model-level inertial and topology audit.

Audit item	Verified value
Inertial block mapping	11/11 rigid-body instances mapped to the intended CAD solid index
Active joints	3 force-actuated prismatic joints
Passive/constraint joints	6 revolute joints and 4 spherical joints
Gravity vector	[0, -9.80665, 0] m/s²
Nominal prismatic viscous damping	0 N·s/m
Total rigid-body mass	4.321118564 kg
