# Supplementary material for: Evaluation of a Fuzzy-Supervised PID Controller for a Parallel Rehabilitation Mechanism
Source: Bioengineering (Basel). 2026 Sep 3;13(9):1029. doi: 10.3390/bioengineering13091029 (PMC13603623; doi:10.3390/bioengineering13091029)
Supplement: Supplementary file 1 [file bioengineering-13-01029-s001.zip › Table_S3.pdf]

Table S3. Inverse-/Forward-Dynamics Numerical Consistency Check

Table S3a. Neutral-configuration inverse-dynamics force check.

Neutral-configuration quantity	Actuator 1 [N]	Actuator 2 [N]	Actuator 3 [N]	Interpretation
Gravity compensation / static inverse dynamics	15.221168124	-13.170858905	-18.126806921	q = 0, gravity enabled
Increment caused by Mz = +1 N·m	0.587741379	1.406431248	-4.605645152	External moment at CAD-defined load frame
Gravity + Mz = +1 N·m inverse-dynamics force	15.808909503	-11.764427657	-22.732452073	Applied to the forward-dynamics consistency check

Table S3b. External-load-to-actuator-force increments at the neutral configuration.

External unit-load increment at q = 0	Actuator 1 [N]	Actuator 2 [N]	Actuator 3 [N]
Fx = +10 N	0.006789935	-0.102637815	0.141111185
Fy = +10 N	-3.054557279	-6.123035391	16.395282043
Fz = +10 N	10.734434161	-13.619294529	-3.949142492
Tx = +1 N·m	68.895270350	69.108103248	68.768300472
Ty = +1 N·m	2.218608104	-4.286714747	-1.701240961
Tz = +1 N·m	0.587741379	1.406431248	-4.605645152

Table S3c. Residual-motion consistency check.

Forward-dynamics consistency metric	Observed result
Check duration	0.05 s
Residual actuator displacement	order of 10^{-9} mm
Residual actuator acceleration	order of 10^{-8} mm/s^2
Interpretation	Internal numerical consistency only; not experimental validation
