# Supplementary material for: Evaluation of a Fuzzy-Supervised PID Controller for a Parallel Rehabilitation Mechanism
Source: Bioengineering (Basel). 2026 Sep 3;13(9):1029. doi: 10.3390/bioengineering13091029 (PMC13603623; doi:10.3390/bioengineering13091029)
Supplement: Supplementary file 1 [file bioengineering-13-01029-s001.zip › Table_S4.pdf]

Table S4. Fuzzy-Supervisor Normalized-Input and Gain-Range Audit

Table S4a. Operating-range audit for the sustained Mz = +1 N·m, 30 s case.

Actuator	Max Kp [N/mm]	Mean Kp [N/mm]	Max Kd [N·s/mm]	Mean Kd [N·s/mm]	Max |normalized input|	Clipping	±100 N saturation
1	22.9106	20.4773	0.63637	0.50378	< 0.8	No	No
2	22.7993	20.4507	0.63417	0.50351	< 0.8	No	No
3	22.2139	20.2327	0.62718	0.50259	< 0.8	No	No

Table S4b. Final fuzzy-supervisor and common integral-channel parameters.

Parameter	Final value
Es	1.3 mm
Eds	16.3 mm/s
Normalized input interval	[-1, 1]
Kp0	20 N/mm
Kd0	0.5 N·s/mm
ΔKp,max	4 N/mm
ΔKd,max	0.165 N·s/mm
Admissible Kp(t)	[20, 24] N/mm
Admissible Kd(t)	[0.5, 0.665] N·s/mm
Ki	2 N/(mm·s), fixed
Leaky-integrator parameter λ	0.02 s^{-1}

The operating-range audit documents simulation behavior only and does not replace hardware safety validation.
