# Supplementary material for: Evaluation of a Fuzzy-Supervised PID Controller for a Parallel Rehabilitation Mechanism
Source: Bioengineering (Basel). 2026 Sep 3;13(9):1029. doi: 10.3390/bioengineering13091029 (PMC13603623; doi:10.3390/bioengineering13091029)
Supplement: Supplementary file 1 [file bioengineering-13-01029-s001.zip › Table_S5.pdf]

Table S5. Detailed Robustness and Sensitivity Results

Table S5a. Signed relative changes for synthetic-noise, viscous-damping, combined-nonideality, and amplitude-sensitivity cases.

Sensitivity case	ΔRMSE [%]	Δmax error [%]	ΔIAE [%]	Mean Δpeak force [%]	Mean ΔRMS force [%]
Noise 0.5×	-0.25974	-0.36257	-0.29053	+0.42859	-0.007
Noise 1×	-0.50124	-0.46163	-0.53054	-0.10874	-0.005
Noise 2×	+0.095398	+1.8668	-0.042225	+0.74132	-0.007
b = 70 N·s/m	-0.20619	+1.0335	-0.21927	-0.004	-0.0004
b = 140 N·s/m	-0.21226	-0.25835	-0.22133	-0.006	-0.0006
b = 280 N·s/m	-0.36112	-0.47590	-0.33428	-0.007	-0.0009
Noise 1× + b = 140 N·s/m	-0.374	-0.415	-0.333	-0.017	-0.001
Amplitude 0.5°	-0.11786	-0.16205	-0.10195	-0.001	—
Amplitude 1.0°	-0.25041	-0.37084	-0.21228	-0.00442	—
Amplitude 1.5°	-0.41975	-6.049	-0.32573	-0.012	—

The disproportionately larger Δmax reduction reported for the 1.5° amplitude case reflects that the fuzzy supervisor raises Kp and Kd in response to the instantaneous tracking error and error rate, concentrating its benefit on peak-error suppression; this effect grows relative to the averaged ΔRMSE and ΔIAE reduction as the amplitude, and hence the peak tracking demand, increases.

Table S5b. Detailed core external-load tracking and force results.

External-load case	Mean RMSE PID [mm]	Mean RMSE FSPID [mm]	ΔRMSE [%]	Mean max PID [mm]	Mean max FSPID [mm]	Δmax [%]	ΔIAE [%]	Global peak force PID/FSPID [N]
Mz = +1 N·m	0.22668	0.22293	-1.5591	0.95001	0.85062	-10.201	-0.60473	39.090 / 39.625
Fy = +10 N	0.18847	0.18445	-1.2922	0.51154	0.46058	-9.3268	-0.54947	22.667 / 22.045
Fz = +10 N	0.27383	0.26582	-3.1505	1.1176	0.99867	-10.068	-0.79028	47.275 / 48.119

Negative tracking changes indicate a lower FSPID error metric than PID. Synthetic-noise values are numerical perturbations, and damping values are parametric model coefficients rather than measured sensor or friction characteristics.
