# Supplementary material for: Evaluation of a Fuzzy-Supervised PID Controller for a Parallel Rehabilitation Mechanism
Source: Bioengineering (Basel). 2026 Sep 3;13(9):1029. doi: 10.3390/bioengineering13091029 (PMC13603623; doi:10.3390/bioengineering13091029)
Supplement: Supplementary file 1 [file bioengineering-13-01029-s001.zip › Table_S6.pdf]

Table S6. Reproducibility-File Inventory

Table S6. Reproducibility-file inventory.

File	Category	Role	Essential?	Notes
Assem_Sim_FSPID_A1.slx	Model	Authoritative forward-dynamics PID/FSPID master	Yes	Configure in memory with A1_setup.m; do not overwrite the disk master
Assem_Sim_inverse_dynamics.slx	Model	Corrected inverse-dynamics / kinematics master	Yes	Use corrected inertial mapping only
Assem_Sim_V2_DataFile.m	CAD/Simscape data	Rigid transforms, masses, CoM, MoI and PoI	Yes	Source for Table S2
FSPID_A1.fis	Controller	Final zero-order Sugeno FIS	Yes	5 × 5 rule base; 25 rules
FSPID_A1_parameters.mat	Controller	Final controller and normalization parameters	Yes	Parameter snapshot
A1_setup.m	Launcher	Reloads untouched master and configures PID/FSPID scenarios in memory	Yes	Clean-restart workflow
A1_CAD_reference.mat	Reference data	Nominal CAD-derived q, qdot and qddot reference	Yes	1.0° Rotation.y, 0.2 Hz
generate_reference_A1_CAD.m	Script	Inverse-kinematic reference generation and verification	Yes	Branch-continuity and closure checks
inertial_mapping_AFTER_fix.csv	Audit	Final inertial mapping audit	Recommended	11/11 entries OK
workspace_sweep_A1_summary.csv	Audit	Local workspace/Jacobian sweep summary	Recommended	Numerical mechanism audit
A1_inverse_dynamics_baseline.mat	Result	Inverse-dynamics gravity baseline	Recommended	Static force histories
A1_FSPID_all3_CAD_nominal_FULL.mat	Result	Full nominal FSPID result	Recommended	Nominal controller signals
A1_FSPID_all3_Mz1_30s.mat	Result	Sustained Mz = +1 N·m FSPID result	Recommended	Operating-range audit
A1_PID_Ki2_CAD.mat	Result	Nominal fixed-gain PID result	Recommended	Matched baseline
